# Inertial-Robotic Motion Tracking in End-Effector-Based Rehabilitation Robots

**DOI:** 10.3389/frobt.2020.554639

**Published:** 2020-11-27

**Authors:** Arne Passon, Thomas Schauer, Thomas Seel

**Affiliations:** Control Systems Group, Technische Universität Berlin, Berlin, Germany

**Keywords:** end-effector-based robots, inertial measurement units, sensor fusion, posture biofeedback, real-time tracking, rehabilitation robots, compensation motion detection, upper-limb rehabilitation

## Abstract

End-effector-based robotic systems provide easy-to-set-up motion support in rehabilitation of stroke and spinal-cord-injured patients. However, measurement information is obtained only about the motion of the limb segments to which the systems are attached and not about the adjacent limb segments. We demonstrate in one particular experimental setup that this limitation can be overcome by augmenting an end-effector-based robot with a wearable inertial sensor. Most existing inertial motion tracking approaches rely on a homogeneous magnetic field and thus fail in indoor environments and near ferromagnetic materials and electronic devices. In contrast, we propose a magnetometer-free sensor fusion method. It uses a quaternion-based algorithm to track the heading of a limb segment in real time by combining the gyroscope and accelerometer readings with position measurements of one point along that segment. We apply this method to an upper-limb rehabilitation robotics use case in which the orientation and position of the forearm and elbow are known, and the orientation and position of the upper arm and shoulder are estimated by the proposed method using an inertial sensor worn on the upper arm. Experimental data from five healthy subjects who performed 282 proper executions of a typical rehabilitation motion and 163 executions with compensation motion are evaluated. Using a camera-based system as a ground truth, we demonstrate that the shoulder position and the elbow angle are tracked with median errors around 4 cm and 4°, respectively; and that undesirable compensatory shoulder movements, which were defined as shoulder displacements greater ±10 cm for more than 20% of a motion cycle, are detected and classified 100% correctly across all 445 performed motions. The results indicate that wearable inertial sensors and end-effector-based robots can be combined to provide means for effective rehabilitation therapy with likewise detailed and accurate motion tracking for performance assessment, real-time biofeedback and feedback control of robotic and neuroprosthetic motion support.

## 1. Introduction

### 1.1. Motivation and Background

Spinal cord injury or stroke can lead to movement disorders like a paresis of the upper limb (Gowland et al., 1992; Popovic and Sinkjaer, 2000). As a result, patients are often gravely impaired in activities of daily living for the rest of their lives. Primary objectives during rehabilitation training are the enhancement of patients' health situation and self-sufficiency. Stroke patients can often additionally benefit from regained motor functions due to the therapy. Robot-assisted rehabilitation and Functional Electrical Stimulation (FES) are well-known technologies and popular means for enhancement of the physical therapy in modern rehabilitation settings (Oujamaa et al., 2009; McCabe et al., 2015). These systems actively support patients during motions that they cannot perform sufficiently well or not often enough without support.

The role of sensor systems in such rehabilitation systems is 3-fold:

1. Feedback control is commonly used to adjust the motion support to the individual patient in real time and thereby enable the execution of accurate movements (Marchal-Crespo and Reinkensmeyer, 2009; Schauer, 2017). This requires sufficiently precise sensor systems that yield real-time measurements of the currently conducted motion.
2. At the same time, such sensor systems facilitate objective recording and assessment of the patients' motor performance, such as speed of execution, completion of tasks and reaction times (Oña et al., 2018).
3. A third major advantage of motion tracking in rehabilitation systems is that it enables biofeedback that informs the patients about their own motion and positive or negative aspects of that motion and their performance (Zhi et al., 2018), for example in a virtual reality environment. While such a biofeedback facilitates gamification of the rehabilitation tasks (Novak et al., 2014), it is also of crucial importance when the patients perform undesired compensatory motions, which means they compensate weakness of the to-be-trained joint or muscle by exaggerated or unphysiological motions of other joints or muscles (Ma et al., 2019).

In upper limb motion, for example, a decreased range of motion of the shoulder and/or elbow joint is often compensated by movement (flexion, inclination, translation) of the upper body or, with the upper body fixed, by movement of the shoulder girdle (Liu et al., 2013; Grimm et al., 2016; Levin et al., 2016). Both movements are possible, both facilitate the desired movement in an undesirable way. The trunk is, for example, moved forward to reach an object instead of extending the arm (Robertson and Roby-Brami, 2011). Preventing compensation during rehabilitation training improves the therapy outcome and decreases long-term problems, such as pain, orthopedic illnesses and learned non-use (Levin et al., 2009). In reaching tasks, moderately to severely impaired patients exhibit mean shoulder displacements of 14 cm, while these displacements are only around 4 cm in healthy subjects (Cirstea and Levin, 2000). An automatic biofeedback that prevents compensatory motion requires a real-time motion tracking solution that is sufficiently precise to distinguish these levels of shoulder displacement due to upper-body or shoulder girdle movements.

### 1.2. Motion Tracking in Exoskeletons vs. End-Effector-Based Robots

**Figure 1:**
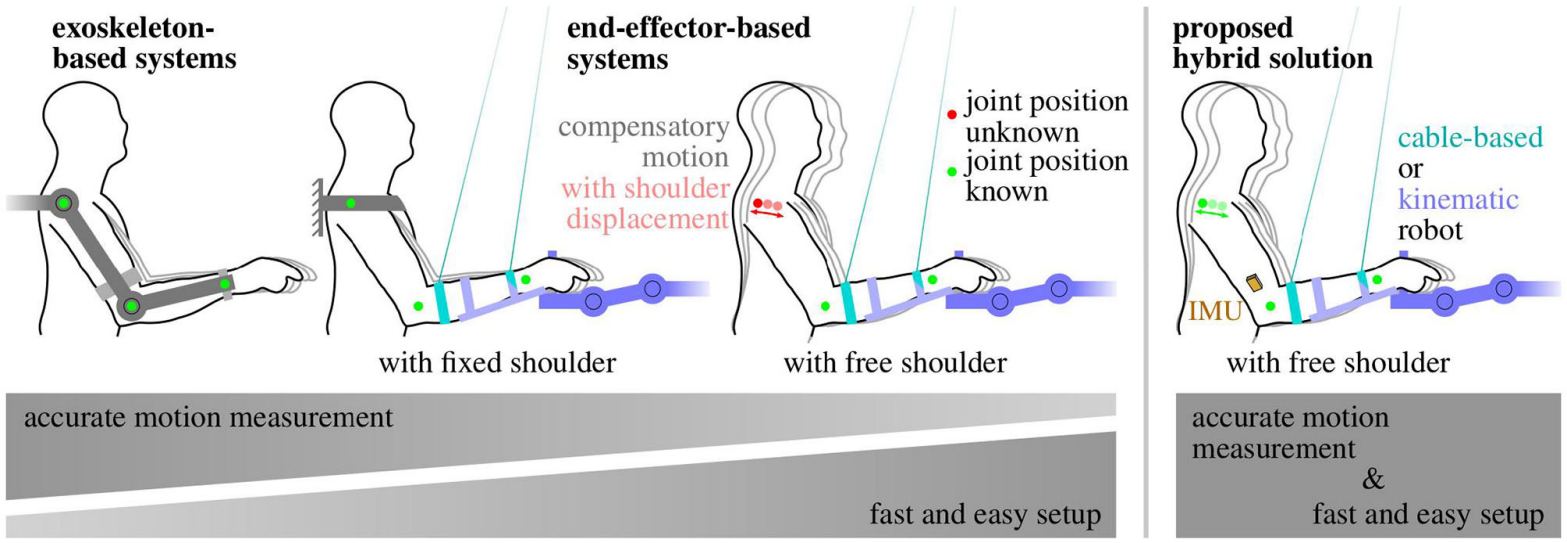
Trade-off between the amount of arm motion measurement information and system complexity in rehabilitation robots. The easier setup of end-effector-based robotic systems comes at the cost of reduced measurement information. The proposed hybrid solution (right) combines both advantages.

In robot-assisted rehabilitation training of the upper limb, a range of different rehabilitation systems with different motion tracking solutions are available or have been proposed (Vito et al., 2014). They can be subdivided into two main groups: (1) exoskeleton-based systems and (2) end-effector-based systems (Maciejasz et al., 2014). The amount of inherently available measurement information decreases from exoskeletons to end-effectors, as illustrated in Figure 1 and detailed in the following.

Exoskeletons reproduce the kinematic structure of the limb they are attached to. The joints of the human limb are assisted and moved by the corresponding joints of the exoskeleton, which also provide measurement information by means of built-in sensors. Major drawbacks of exoskeletons are that they are quite obtrusive and must be adjusted precisely to the individual segment lengths and joint axes, which can be time-consuming (Maciejasz et al., 2014). A misalignment can even cause unwanted pain and in the worst case long-term damage (Sicuri et al., 2014; Bertomeu-Motos et al., 2018). Besides that, the estimation of the human arm joint angles from the exoskeleton ones is often non-trivial if their kinematic structures differ (Nordin et al., 2014). Therefore, rather complex solutions have been proposed, such as extended inverse kinematics posture estimation (EIKPE) models (Wu et al., 2015; Cortés et al., 2016). Some exoskeletons even self-align the robot's active rotational axes to the user's joint axes by means of passive rotational joints, which further increases kinematic complexity (Trigili et al., 2019). If the exoskeleton is well-adjusted to the subject and a transformation of its kinematics to the anatomical frames is available, good measurements of the subject's arm pose and joint angles can be obtained (Nordin et al., 2014).

End-effector-based systems are robotic systems that are only attached to the distal segments of the limbs, which is typically realized by a handle for the hand, a forearm brace, or a footplate. A subgroup of end-effector-based systems are cable-driven motion support robots, which use ropes to provide gravitation-compensating and motion-promoting forces to a distal limb segment. Compared to exoskeletons, end-effector-based systems require far less adjustment to individual patients (Burgar et al., 2000; Lum et al., 2002). However, as a direct consequence of the reduced contact between human and robotic system, only the motion of one body segment is measured by the robot, and the motion of all adjacent segments must be inferred using mechanical models (Nordin et al., 2014) or additional sensors. For end-effector-based upper limb rehabilitation systems, which yield direct measurement of a distal segment, the motion of the upper arm is commonly inferred using the simple assumption of a fixed shoulder position (Dipietro et al., 2007; Rosati et al., 2007).

Figure 1 summarizes the general observation that an easier setup and positioning of the patient comes at the cost of reduced measurement information and accuracy. This drawback can be compensated if end-effector-based systems are combined with wearable sensor technology that ideally requires only little setup effort.

The conventional gold standard for human motion assessment are multi-camera systems that track a set of reflective or active markers that are worn on anatomical landmarks. However, these systems are expensive, and they require a complex marker and camera setup as well as a clear line-of-sight between each marker and at least two cameras at all times (Kirk et al., 2005; Zhou and Hu, 2008). Other optical systems, such as depth cameras (e.g., Microsoft Kinect) or single cameras have not reached comparable accuracy due to occlusion, jitter, low and varying sampling frequency or viewing-angle-dependent performance deterioration (Yahya et al., 2019).

Body-worn goniometers overcome the line-of-sight restrictions and can yield root-mean-squared error values (RMSE) around 2° (Tognetti et al., 2015). However, they are obtrusive in the sense that they span across joints, and they only measure joint angles but neither orientations nor velocities nor positions (Tognetti et al., 2015).

Inertial measurement units (IMUs) are wearable sensors that provide all this information without requiring cables or clear lines of sight between the sensors (Held et al., 2018). However, IMUs require, in general, more complex sensor fusion algorithms to address magnetic disturbances, integration drift, and sensor-to-segment misalignment. Each IMU is composed of three types of sensors (gyroscopes, accelerometers, and magnetometers) and measures the three-dimensional angular rate, acceleration, and magnetic field vector in its intrinsic coordinate system. IMUs are small and lightweight enough to be considered completely unobtrusive and assure zero influence on the motion performance. Recent advances in inertial motion tracking have helped to overcome long-standing challenges, such as sensor-to-segment calibration (Taetz et al., 2016; Nowka et al., 2019; Olsson et al., 2019) or the requirement of a homogeneous magnetic field (Laidig et al., 2017b; Laidig et al., 2019; Seel and Ruppin, 2017) and to provide a similar accuracy as optical systems (Seel et al., 2015; Filippeschi et al., 2017; Salchow-Hömmen et al., 2019).

### 1.3. State of the Art in Sensor Systems for End-Effector-Based Upper Limb Therapy

Since the application-related focus of the present work lies on upper-limb rehabilitation robots, we also briefly review existing systems and solutions for this specific application domain. A comprehensive survey of upper-limb rehabilitation systems is given in Maciejasz et al. (2014) and Mekki et al. (2018). We focus more specifically on systems and methods that combine end-effector-based motion support systems with wearable or optical motion tracking solutions to compensate the lack of measurement information of the former by means of the latter. Below we provide an overview of existing combinations and of their solutions for compensatory motion detection.

For end-effector-based rehabilitation robots, two main sensor setups have been proposed: on the one hand the combination with a depth camera and on the other hand combinations with inertial sensors or solely accelerometers.

Regarding camera-based solutions, Brokaw et al. (2013) combined a wrist brace robot with a Kinect (Microsoft, USA) sensor to calculate the trunk and arm joint angles during reaching motions and demonstrated that these angles can be used to prevent compensatory movements. However, they reported large tracking errors of the Kinect due to occlusion and problems to distinguish between the subject's arm and the robot. In a similar work, Zhi et al. (2018) recently published results on compensatory motion classification based on Kinect's skeletal tracking information. Occlusion occurred, and a solution for posture biofeedback was not presented. Another approach using a Kinect and two end-effectors for each arm was evaluated by Valdés and der Loos (2017). They detected trunk compensation by measuring motions of the shoulder-spine joint using the Kinect. Two biofeedback strategies were compared, both of which were shown to reduce compensatory movements. However, yet again a continuous clear line-of-sight is required, which is especially a problem if the therapist has for any reason to act on the patient.

Mihelj (2006) presented the combination of a hand-attached robot with two accelerometers at the upper arm. This approach yields accurate upper-limb joint angles. However, the inverse-kinematics algorithm requires shoulder joint fixation, for example by binding the trunk to a chair with belts, which leads to additional setup effort. Bertomeu-Motos et al. (2015b) further improved the method by Mihelji proposing only the use of one accelerometer at the upper arm, but also for this method the shoulder position must be known and fixed during the therapy. A similar yet even more restrictive approach, which uses a forearm cuff that prevented forearm pronation-supination and wrist movements, is found in Papaleo et al. (2015) and is also used by Scotto di Luzio et al. (2018).

To our best knowledge, the only combination of an end-effector-based robot with inertial sensors that does not require shoulder fixation was proposed by Bertomeu-Motos et al. (2015a) and validated in stroke patients (Bertomeu-Motos et al., 2018). The elbow angle and shoulder position are estimated using inertial sensors on the upper arm (only accelerometer) and on the outer edge of the shoulder, with accuracies below 6° and 5 cm, respectively. However, the proposed algorithms require two sensor units, and they rely on magnetometer readings, which implies that they are unreliable if the earth-magnetic field is disturbed, such as in indoor environments, near ferromagnetic material or electronic devices, i.e., practically in all realistic clinical settings (de Vries et al., 2009; Le Grand and Thrun, 2012; Subbu et al., 2013; Shu et al., 2015; Salchow-Hömmen et al., 2019). Furthermore, compensatory motion detection or biofeedback has not been considered in that article.

In a previous work, we combined a cable-driven end-effector-based robot with magnetometer-free inertial sensors worn on the forearm and upper arm (Passon et al., 2018). We demonstrated that fusing cuff position measurements of the robotic system with inertial sensor readings is advantageous and enables magnetometer-free tracking of the complete forearm orientation and position. For temporary compensatory displacements of the trunk or shoulder (less than a half minute), we were able to estimate the upper arm heading and the true shoulder position accurately. However, the approach failed to provide long-time stable estimates under longer lasting compensation movements or static compensatory postures.

In summary, depth-camera-based solutions can lead to reliable compensation motion detection but only under continuous line-of-sight restrictions. These restrictions can be overcome by means of wearable inertial sensors, but there is a lack of practical solutions that provide long-time stable motion tracking of the entire upper limb in realistic environments with inhomogeneous magnetic fields. To date there is no inertial sensor-based solution that yields all of the following desirable features:

(1) Accurate measurement information of the complete orientation of the upper arm independent of the local magnetic field;
(2) Reliable long-time stable real-time detection of shoulder displacements and associated compensatory motion.

### 1.4. Contributions of the Paper

**Figure 2:**
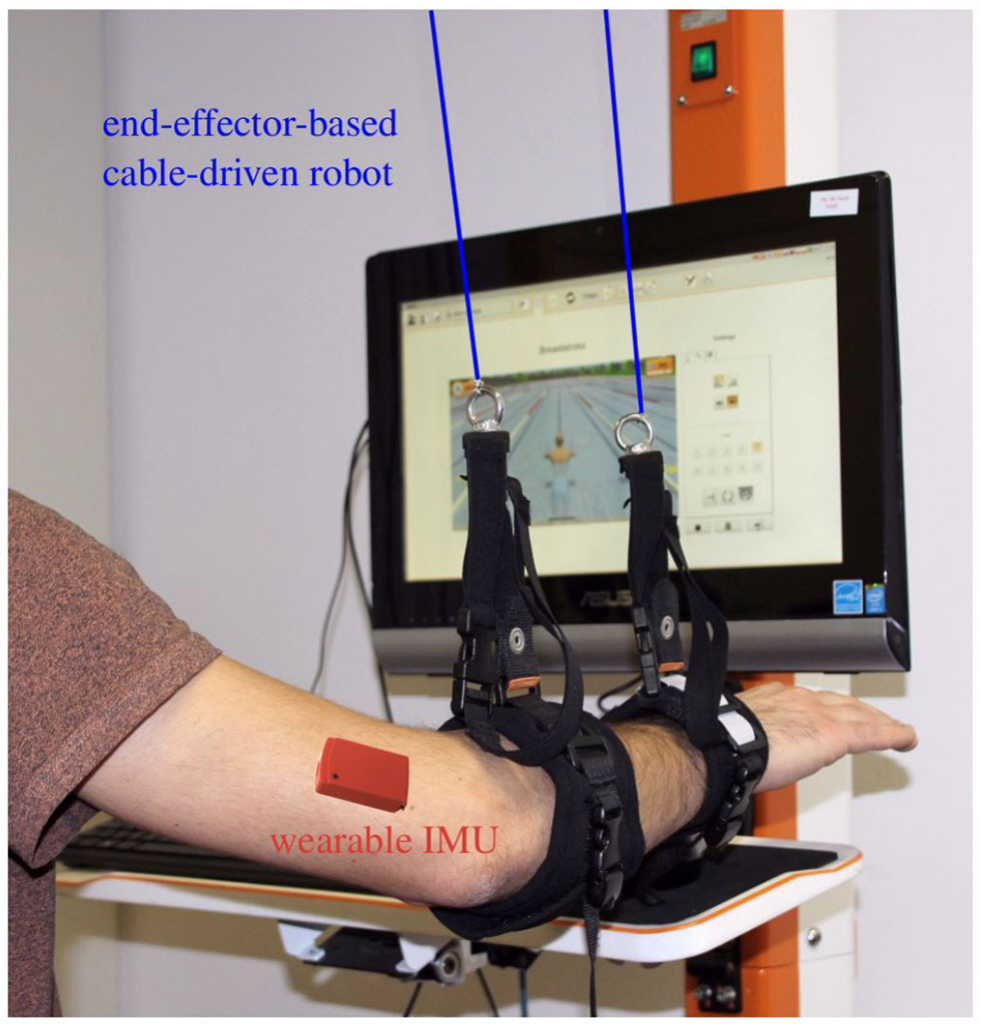
Example setup: the cable-based rehabilitation robot Diego (Tyromotion GmbH, Austria) is augmented by one wearable inertial sensor at the upper arm to enable tracking of the upper arm and shoulder motion.

In order to address the aforementioned challenges, we propose new methods that leverage the full potential of combining end-effector-based rehabilitation robots with wearable inertial sensors. We will demonstrate in one particular experimental setup that the measurement limitations of an end-effector-based robot can be overcome by exploiting inertial measurements. Simultaneously, fundamental limitations of inertial motion tracking are overcome by a novel magnetometer-free sensor fusion method that exploits the end-effector-based measurements. In brief, the main contributions of this paper are:

1. We introduce sensor fusion methods that combine robotic measurements from one segment and inertial measurements of an adjacent segment to determine long-time stable measurements of the full orientation and the endpoint of that adjacent segment in the robotic measurement frame while
  - using *no* magnetometer readings at all,
  - allowing the adjacent segment to move freely,
  - and requiring no initial heading alignment or position calibration.
2. We apply the proposed methods to augment the cable-based forearm-attached robot shown in Figure 2 with a wearable IMU and determine long-time stable measurements of the upper arm orientation and shoulder position in real time.
3. We perform an experimental validation with five subjects and a camera-based ground truth measurement, and we demonstrate that the achieved measurement accuracy is around 4° and 4 cm, respectively.
4. We propose a method that detects compensatory motions with shoulder displacements greater ±10 cm that last for more than 20% of a motion cycle, and we demonstrate that typical rehabilitation motions conducted by healthy subjects with and without compensatory motion are 100% correctly classified by this method.

The remaining paper is structured as follows. The general hardware setup and problem statement are presented in Section 2. The developed methods for sensor fusion, shoulder position estimation and detection of compensation movements are introduced in Section 3. The experimental procedure including a description of the rehabilitation robot that is considered for validation and the analysis of conducted experiments with five healthy subjects is given in Section 4. Finally, a discussion of the results is presented in Section 5, and conclusions follow in Section 6.

## 2. Kinematic Model and Sensor Fusion Task

Before defining the sensor fusion task, we describe the general properties and assumptions of the kinematic system and possible example realizations. Consider a kinematic chain consisting of two rigid segments, and let both be connected by a joint with up to three rotational degrees of freedom.

(i) Assume that one of both segments is in contact with a robotic system, and call this segment the *connected* segment and the other one the *adjacent* segment.
(ii) The robot is assumed to yield real-time information of the position of the joint between both segments in a fixed robotic coordinate system.
(iii) The robot, however, yields no information on the orientation of the adjacent segment, and we also *refrain* from assuming that any point along that segment remains fixed in space.

Two examples are given to illustrate the relevance of this general kinematic system and the meaning of the assumptions. In the first example, the connected and adjacent segments are the shank and thigh, respectively. A cable-based robotic system with above-ankle and below-knee cuffs measures the cuff positions and determines the knee position by extrapolating the line between both cuffs. The orientation of the thigh, however, cannot be determined if we refrain from assuming a fixed hip position. In the second example, a robotic system connects to the upper extremity via a forearm brace, which enables measurements of the orientation and position of the forearm and elbow but not the motion of upper arm and the unconstrained shoulder. In both examples, the adjacent segment is the proximal one of both segments, which makes sense in the context of end-effector-based robots. However, it should be noted that neither the kinematic model nor the methods we will propose are limited to that case.

**Figure 3:**
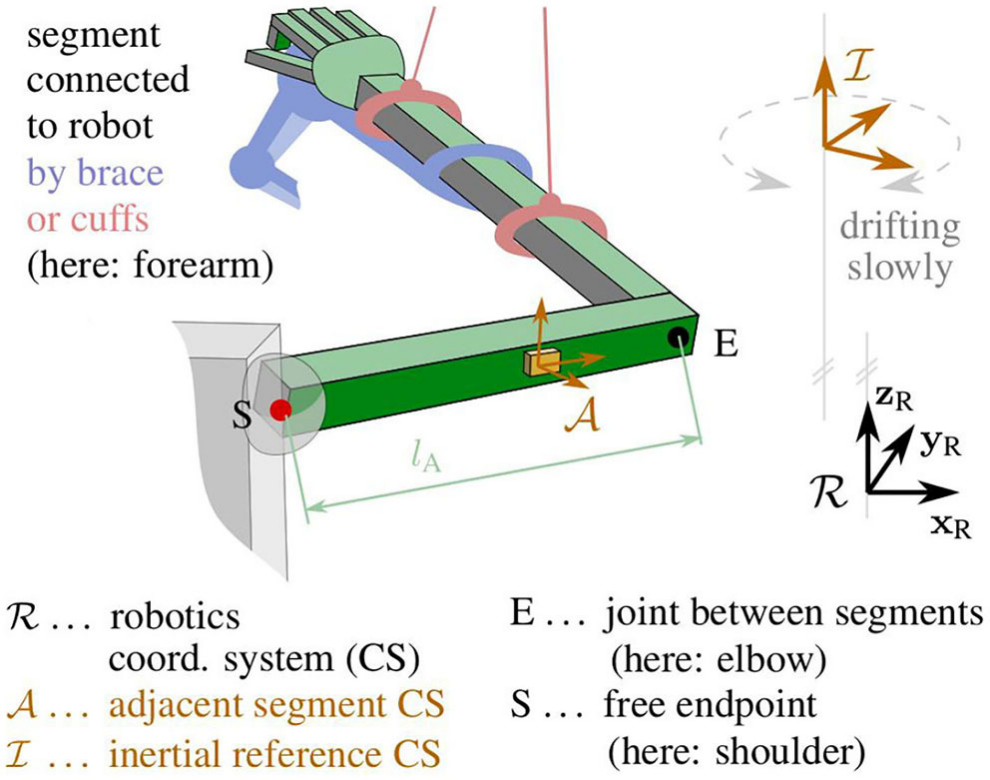
Kinematic model of the upper extremity with definitions of the robotic frame R, the intrinsic IMU frame A, the inertial reference frame I, and the joints E and S.

To obtain complete measurement information of the motion of both segments, a wireless wearable IMU is attached to the adjacent segment. Figure 3 shows one specific example of such a hybrid inertial-robotic measurement system for the upper extremity. We assume that the distance of the sensor to the joint is approximately known. We further assume that the relative orientation between the inertial sensor and the segment is known either by careful sensor-to-segment attachment or by employing methods that automatically determine this information from almost arbitrary movements of the kinematic system (Müller et al., 2016; Laidig et al., 2017a; Olsson et al., 2019).

The IMU yields three-dimensional measurements of the acceleration, the angular rate and the magnetic field vector in its own intrinsic coordinate system. Since these measurements are obtained from micro-electro-mechanical systems (MEMS), they are prone to considerable bias and noise errors. Nevertheless, the orientation of the sensor can be determined with respect to an inertial frame of reference with a vertical axis and a horizontally northbound axis. This is a standard problem in inertial sensor fusion, and several suitable orientation estimation algorithms exists.

However, two issues arise: Firstly, the inertial reference is not aligned with the robotic coordinate system—only the vertical axes of both frames coincide. Secondly, IMU-based orientation estimation requires a homogeneous magnetic field and therefore fails in indoor environments and in the proximity of ferromagnetic material and electronic devices. In the realistic case of a disturbed and inhomogeneous magnetic field, only the inclination but not the heading of the sensor can be determined reliably.

We conclude that neither the robotic system nor the inertial sensor yields the desired orientation and position information of the adjacent segment. Only the inclination of the segment and the position of one of its ends can be determined. The task that should be addressed in the following is to determine the full orientation and position of the segment by fusing the robotic and inertial measurements.

For the specific example application of a forearm-connected robotic system, we deduce accuracy requirements for the hybrid system to detect the shoulder displacements that are characteristic for compensatory motions in upper-limb rehabilitation of neurological patients: to reliably distinguish between the aforementioned physiological and compensatory shoulder displacement amplitudes [4 vs. 14 cm (Cirstea and Levin, 2000)] during one motion, a method should ideally be able to track the shoulder position with a tracking error of at most 5 cm in average over the course of that motion.

## 3. Proposed Methods

The novel methods are presented in three steps: First we will propose methods that solve the given sensor fusion task for the general kinematic system and determine the orientation of the adjacent segment. We will then demonstrate how the joint angle and the adjacent segment's endpoint position can be calculated from the estimated segment orientation. Finally, we will consider the specific application of upper-limb rehabilitation and propose methods for distinguishing proper motions from motion with undesirable shoulder displacements.

### 3.1. Coordinate Systems and Notation

Let the right-handed robotic workspace coordinate system {**x**_R_, **y**_R_, **z**_R_} be defined by the y-axis **y**_R_ pointing horizontally forward and the z-axis **z**_R_ straight up, as illustrated in Figure 3. The adjacent segment's coordinate system {**x**_A_, **y**_A_, **z**_A_}, in which the inertial measurements are taken, is defined with the x-axis **x**_A_ being parallel to the longitudinal axis of the segment and pointing toward the joint with the connected segment. The inertial reference coordinate system of the orientation estimates is denoted {**x**_I_, **y**_I_, **z**_I_}. The reference frame I has a vertical z-axis but horizontal x- and y-axes with an arbitrary, slowly drifting heading, as will be explained later on.

While the lower right index is used to denote to which coordinate system a vector is attached, the lower left index is used to describe in which coordinate systems it is expressed. For example, **x**_A_ denotes the x-axis of the coordinate system A; ${\;}_{A}^{}{\text{x}}_{\text{A}}$ denotes the coordinates of that x-axis in the very same frame, which trivially and constantly is [1, 0, 0]⊺; finally, ${\;}_{R}^{}{\text{x}}_{\text{A}}$ denotes the coordinates of that x-axis in the robotic workspace frame R. For quaternions, the upper and lower left indices are defined such that ${\;}_{R}^{A}\text{q}$ is the quaternion that fulfills $\left[0,{\;}_{R}^{}\text{v}⊺\right]⊺={\;}_{R}^{A}\text{q}⊗\left[0,{\;}_{A}^{}\text{v}⊺\right]⊺⊗{\;}_{A}^{R}\text{q}$ for any vector ${\;}_{}^{}\text{v}\in {\mathbb{R}}^{3}$. Note that the abbreviated notation ${\;}_{R}^{}\text{v}={\;}_{R}^{A}\text{q}⊗{\;}_{A}^{}\text{v}⊗{\;}_{A}^{R}\text{q}$ is used in the further course of this work, i.e., we assume that the quaternion multiplication operator ⊗ regards three-dimensional vectors automatically as their pure quaternion counterpart. Finally, we define the operator [·]_normalize_ that maps any vector to a vector with the same direction but Euclidean norm one.

### 3.2. Estimation of the Adjacent Segment's Orientation

We estimate the orientation of the adjacent segment with respect to an inertial reference frame by employing a quaternion-based sensor fusion algorithm that uses strapdown integration of the angular rates and geodetic accelerometer-based corrections (Seel and Ruppin, 2017). Note that, while the algorithm is capable of also performing magnetometer-based corrections, we refrain from using the magnetometer readings and only fuse gyroscope and accelerometer measurements.

Denote the raw accelerometer readings of the IMU by ${\;}_{A}^{}{\tilde{\text{a}}}_{\text{A}}$ and the raw gyroscope readings by ${\;}_{A}^{}\omega_{\text{A}}$ in the coordinate system of the adjacent segment. Here, $\tilde{\text{a}}$ is the specific force (Titterton et al., 2004), which is the sum of the linear acceleration **a** due to velocity changes and gravitational acceleration. At each sampling instant *t*, the sensor fusion algorithm takes ${\;}_{A}^{}{\tilde{\text{a}}}_{\text{A}}\left(t\right)$, ${\;}_{A}^{}\omega_{\text{A}}\left(t\right)$ and provides the quaternion ${\;}_{I}^{A}\text{q}$ that describes the orientation of the segment frame A with respect to the reference frame I.

This orientation estimate has reliable and accurate inclination components, but the heading is unknown in the following sense: The reference frame has a vertical z-axis but an arbitrary heading that depends on the initial orientation of the IMU and the initial values of the orientation estimation filter. Moreover, that heading of the reference frame is drifting slowly due to gyroscope bias and integration drift, which implies that there is an unknown and slowly drifting heading offset δ between the frames I and R.

In the present contribution, we demonstrate that this missing heading information can be inferred if the position ${\;}_{R}^{}{\text{p}}_{\text{E}}$ of the joint in the robotic frame is known. We will estimate the heading of the adjacent segment by exploiting the kinematic relation between the acceleration measured by the inertial sensor and the numerically determined second time derivative of ${\;}_{R}^{}{\text{p}}_{\text{E}}$.

#### 3.2.1. Joint Acceleration Disagreement

We use the approximately known distance ${\;}_{A}^{}{\text{p}}_{\text{E}}$ between the IMU and the joint to determine an IMU-based estimate of the joint acceleration **a**_E_ in the inertial reference frame. For this purpose, we first determine the specific force ${\tilde{\text{a}}}_{\text{E}}$ in the A-frame by accounting for the radial and tangential acceleration due to rotation around the joint:

(1) $$\begin{array}{r} {\;}_{A}^{}{\tilde{\text{a}}}_{\text{E}}={\;}_{A}^{}{\tilde{\text{a}}}_{\text{A}}+{\left({\left[{\;}_{A}^{}\omega_{\text{A}}\right]}_{\times }\right)}^{2}{\;}_{A}^{}{\text{p}}_{\text{E}}+{\left[{\;}_{A}^{}{\overset{∙}{\omega }}_{\text{A}}\right]}_{\times }{\;}_{A}^{}{\text{p}}_{\text{E}}, \end{array}$$

where [·]_×_ denotes the cross product matrix, and the time derivative ${\;}_{A}^{}{\overset{∙}{\omega }}_{\text{A}}$ is determined by numerical differentiation of the low-pass filtered angular rate (5th-order Butterworth filter with a cutoff frequency of 2.5 Hz). We then use the orientation quaternion ${\;}_{I}^{A}\text{q}$ to transform the specific force into the inertial reference frame and to remove the gravitational acceleration to obtain the acceleration of the joint:

(2) $$\begin{array}{r} {\;}_{I}^{}{\text{a}}_{\text{E}}={\;}_{I}^{A}\text{q}⊗{\;}_{A}^{}{\tilde{\text{a}}}_{\text{E}}⊗{\;}_{A}^{I}\text{q}-\left[0,0,9.81\right]⊺. \end{array}$$

Ideally, ${\;}_{I}^{}{\text{a}}_{\text{E}}$ and the second time derivative ${\;}_{R}^{}{\ddot{\text{p}}}_{\text{E}}$ are the same quantity expressed in different frames (I and R), which only differ in their heading. In practice, however, when transforming both signals into the same frame, we find high frequency deviations caused by noise and soft-tissue motion. Thus, the best we can ask for is that they are similar in the sense of a small sum of squared differences of the low-pass filtered signals (5th-order Butterworth filter with a cutoff frequency of 0.5 Hz).

Denote the unknown heading offset between the frames R and I by δ(*t*) ∈ [0, 2π) and recall that it is an unknown but only slowly drifting angle. For any given value of δ and any moment in time, we can determine the disagreement *d*(δ):[0, 2π) → ℝ between a given value of ${\;}_{I}^{}{\text{a}}_{\text{E}}$ and a given value of ${\;}_{R}^{}{\ddot{\text{p}}}_{\text{E}}$ by

(3) $$\begin{array}{r} d\left(\delta ,{\;}_{I}^{}{\text{a}}_{\text{E}},{\;}_{R}^{}{\ddot{\text{p}}}_{\text{E}}\right):=\left[\begin{array}{c} \cos\left(\frac{\delta }{2}\right)\\ 0\\ 0\\ \sin\left(\frac{\delta }{2}\right) \end{array}\right]⊗{\;}_{I}^{}{\text{a}}_{\text{E}}⊗\left[\begin{array}{c} \cos\left(\frac{\delta }{2}\right)\\ 0\\ 0\\ -\sin\left(\frac{\delta }{2}\right) \end{array}\right]-{\;}_{R}^{}{\ddot{\text{p}}}_{\text{E}}. \end{array}$$

Let the cost function *c*(δ, *t*):[0, 2π) × ℝ^+^ → ℝ be the sum of the squares of this disagreement over a moving window:

(4) $$\begin{array}{r} c\left(\delta ,t\right):=\overset{m}{\underset{k=1}{\sum }}d{\left(\delta ,{\;}_{I}^{}{\text{a}}_{\text{E}}\left(t-kTs\right),{\;}_{R}^{}{\ddot{\text{p}}}_{\text{E}}\left(t-kTs\right)\right)}^{2}, \end{array}$$

where *m* is the width of the moving window. Finally, define a grid-based optimization function optdelta(Δ, *t*) that takes a given moment *t* > *mTs* and a given set Δ of heading offset values and returns the value from the grid-set that minimizes the disagreement over the time window [*t* − *mTs,t*]:

(5) $$\begin{array}{r} \text{optdelta}\left(\Delta ,t\right)=\underset{\delta \in \Delta }{\text{arg min}}\left(c\left(\delta ,t\right)\right). \end{array}$$

#### 3.2.2. Relative-Heading Estimation

The slowly drifting relative heading δ, and thus the heading of the adjacent segment in the robotic workspace frame R, is determined by the following algorithm.

**Figure 4:**
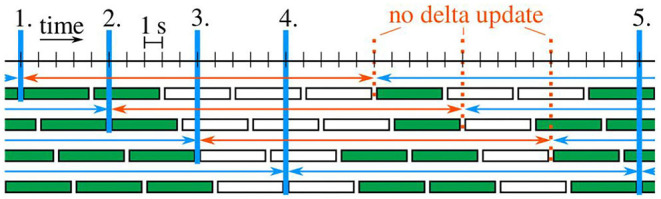
Example case with five delta updates (blue verticals). Sub-windows are filled green if enough considerable motion is contained. Each time window with at least three green sub-windows is marked by a blue (otherwise red) arrow.

Every 5 s, a time window containing the last 20 s of data is considered, and that time window is split into five sub-windows (see Figure 4). For each sub-window, we say that there is no considerable motion if the elbow position ${\;}_{R}^{}{\text{p}}_{\text{E}}$ remains within a sphere of ten-centimeters diameter throughout the 4 s. If at least three sub-windows of the considered time window contain considerable motion, then the entire window is said to contain enough considerable motion.

Initially, the estimated heading offset $\hat{\delta }$ is undefined. At each of the first five time windows that contain enough considerable motion, the estimate is updated by the following two-steps:

(6) $$\begin{array}{r} \tilde{\delta }=\text{optdelta}\left(\left\{0^{{}^{\circ}},5^{{}^{\circ}},\ldots ,355^{{}^{\circ}}\right\},t\right), \end{array}$$

(7) $$\begin{array}{r} \hat{\delta }=\text{optdelta}\left(\left\{\tilde{\delta }-5^{{}^{\circ}},\tilde{\delta }-4^{{}^{\circ}},\ldots ,\tilde{\delta }+5^{{}^{\circ}}\right\},t\right). \end{array}$$

At all subsequent time windows that contain enough considerable motion, the algorithm checks whether the last five estimates each change by at most 5° from one estimate to the next. As soon as this condition is fulfilled, the algorithm is said to have converged, and all following estimates are determined in a one-step update on a reduced grid that is centered around the estimate ${\hat{\delta }}^{-}$ of the previous time window:

(8) $$\begin{array}{r} \hat{\delta }=\text{optdelta}\left(\left\{{\hat{\delta }}^{-}-5^{∙},{\hat{\delta }}^{-}-4^{∙},\ldots ,{\hat{\delta }}^{-}+4^{∙},{\hat{\delta }}^{-}+5^{∙}\right\},t\right). \end{array}$$

Obviously, we could likewise use the two-step update for all time windows with enough considerable motion or we could switch back to that two-step update whenever consecutive results of the one-step update are five degrees apart. However, the experimental results in Section 4.3 will demonstrate that such extensions would be useless in the sense that they are never triggered.

The proposed periodic updates provide new estimates of the heading offset $\hat{\delta }$ every 5 s as long as the adjacent segment moves. When there is almost no motion, the estimate remains constant. Note that the method's accuracy level is directly determined by the user. With the proposed parametrization, the highest accuracy that the algorithm can achieve is 1°, which is more than sufficient for the present application.

### 3.3. Estimation of the Joint Angle and Segment Endpoint Position

We use the estimated heading offset $\hat{\delta }$ between the frames R and I to determine the orientation ${\;}_{R}^{A}\text{q}$ of the adjacent segment in the robotic workspace frame R:

(9) $$\begin{array}{r} {\;}_{R}^{I}\text{q}=\left[\begin{array}{c} \cos\left(\frac{\hat{\delta }}{2}\right)\\ 0\\ 0\\ \sin\left(\frac{\hat{\delta }}{2}\right) \end{array}\right], \end{array}$$

(10) $$\begin{array}{r} {\;}_{R}^{A}\text{q}={\;}_{R}^{I}\text{q}⊗{\;}_{I}^{A}\text{q}. \end{array}$$

By assumption, the longitudinal axis **x**_C_ of the connected segment is known in the robotic workspace frame R. The joint angle can thus be determined as the angle between the longitudinal axes of both segments:

(11) $$\vartheta_{\text{E},\text{hyb}}=∢\left({}_{R}x_{\text{C}},\;{\;}_{R}^{A}q⊗{\;}_{A}x_{\text{A}}{⊗}_{A}^{R}q\right).$$

The orientation ${\;}_{R}^{A}\text{q}$ is further used to calculate the position ${\;}_{R}^{}{\text{p}}_{\text{S}}$ of the endpoint of the adjacent segment from the joint position ${\;}_{R}^{}{\text{p}}_{\text{E}}$ and the segment length *l*_A_:

(12) $$\begin{array}{r} {\;}_{R}^{}{\text{p}}_{\text{S}}={\;}_{R}^{}{\text{p}}_{\text{E}}-{\;}_{R}^{A}\text{q}⊗{\;}_{A}^{}{\text{x}}_{\text{A}}l_{\text{A}}⊗{\;}_{A}^{R}\text{q}. \end{array}$$

For segment lengths of up to half a meter, orientation errors of 1° cause endpoint position errors below 1 cm. Therefore, if position errors in the range of 1 cm are negligible, then a heading estimation accuracy level of 1° is sufficient.

### 3.4. Detection of Compensatory Motions for Biofeedback

In upper-limb rehabilitation, compensatory shoulder motions lead to an inefficient rehabilitation training and can even have a harmful effect for the user. The methods proposed above enable upper arm and shoulder motion tracking by combining end-effector-based robots that connect to the forearm and a wearable IMU on the upper arm.

We propose a method that detects whether the user compensates weakness of affected muscles or joints by trunk or shoulder girdle motions. Without loss of generality, we consider a scenario with periodic arm rehabilitation motions, and we aim at distinguishing the following two cases:

- The *proper movement* (*prop*.*mov*.) in which most of the motion is realized by shoulder joint and elbow joint motions and only minor shoulder displacements around a mean of 4 cm occur (cf. Cirstea and Levin, 2000),
- The *compensatory movement* (*comp*.*mov*.) in which the shoulder and elbow joint remain rather stiff and large portions of the motion are realized by shoulder girdle or trunk motions, which leads to shoulder displacements around a mean of 14 cm (cf. Cirstea and Levin, 2000).

**Figure 5:**
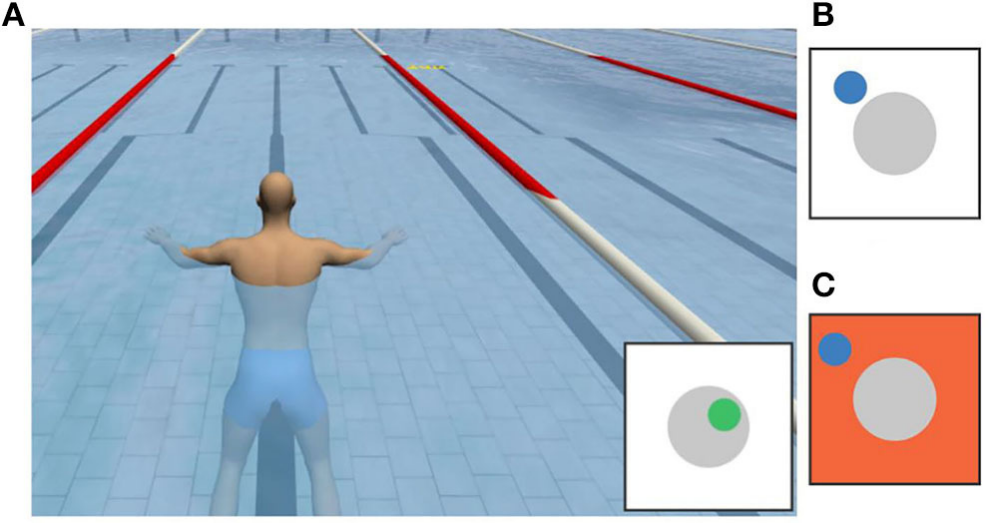
Potential realization of a visual biofeedback: **(A)** rehabilitation game with an indicator that shows a green circle inside the gray tolerance area when the movement is proper. **(B)** Blue circle outside the gray area indicating a large shoulder displacement. **(C)** Orange background indicating a large shoulder displacement for longer than the maximum tolerated duration.

The estimated shoulder position ${\;}_{R}^{}{\text{p}}_{\text{S}}$ is monitored in real-time whether it leaves a tolerated range. An acceptable region of ±10 cm around the therapeutically desired shoulder position covers typical variations during proper arm motions (see Section 4.3). Even healthy subjects sometimes temporarily exceed this limit. In order to tolerate for such short-time deviations, we allowed shoulder displacements of more than ±10 cm for up to 20% of an iteration during repetitive movement training. In applications where such a violation is unacceptable or undesired, these values could of course be lowered. Each iteration is examined when it is completed and detected shoulder displacements are then directly signalized. Whenever such compensatory motions are detected, the user is instructed to move back to the nominal shoulder position and to redo or resume the therapy task. Figure 5 shows one potential implementation of a visual biofeedback. The indicator in the lower right corner of the rehabilitation game shows the tolerated shoulder position region as a gray circle in the center. The estimated current shoulder position is indicated by a smaller circle, which is green during proper movements and turns blue when it is outside the gray tolerance area, i.e., when the shoulder displacement is larger than the preset threshold. If that condition is fulfilled for longer than a short user-defined duration, which might be chosen as a percentage of the current cycle duration, the indicator's background is highlighted in orange to provide a strong warning feedback.

## 4. Experimental Validation

The proposed methods are validated experimentally in a specific application example of robot-assisted upper-limb rehabilitation. We first describe the robotic system, then the experimental procedure, and finally the results.

### 4.1. The Cable-Based Rehabilitation Robot

We consider the cable-based rehabilitation robot Diego (Tyromotion GmbH, Austria), which is an active arm weight compensation and motion support system (Jakob et al., 2018). It facilitates three-dimensional arm therapies that would otherwise be impossible for patients with paretic upper limbs or would require continuous manual support by a therapist. Such motion support can reduce physical fatigue of the patient and therapist and can thereby enable longer therapy sessions. The therapy focus can be on different movements like reaching or lifting or even on the motion of specific joints. The robot is equipped with a virtual game environment to further motivate the patient, even during frequent repetitions of the same movement.

Two retractable ropes are connected to the forearm using one cuff around the wrist and another cuff close to the elbow, as depicted in Figure 2. The rope forces are controlled by independent drives, which also provide measurements of the length of the ropes. Near the outlet of each rope, a low-friction spherical shell moves along with the rope and provides two-dimensional measurement of the rope deflection angle. Combining the length and angle measurements yields estimates of the rope end points, i.e., the forearm cuff positions ${\;}_{R}^{}{\text{p}}_{\text{W}}$ at the wrist and ${\;}_{R}^{}{\text{p}}_{\text{P}}$ of the proximal cuff, in a robot-fixed coordinate system. The elbow position in the robotic workspace frame is calculated by extrapolating the line between both cuff positions into the elbow joint:

(13) $$\begin{array}{r} {\;}_{R}^{}{\text{p}}_{\text{E}}=l_{p}{\left[{\;}_{R}^{}{\text{p}}_{\text{P}}-{\;}_{R}^{}{\text{p}}_{\text{W}}\right]}_{\text{normalize}}+{\;}_{R}^{}{\text{p}}_{\text{P}}, \end{array}$$

where *l*_*p*_ is the known approximate distance between the proximal cuff attachment point and the elbow.

It is important to note that these robotic position measurements are drift-free but provided at a sampling rate of only 25 Hz and susceptible to disturbances by rope oscillations. These disturbances are significant, since the ropes are not always taut—especially when the direction of motion changes suddenly—and small changes of the aforementioned deflection angles have large effects on the estimated endpoint position. In previous research, we have demonstrated that these shortcomings can be overcome by augmenting the robot with a wearable IMU on the forearm, and highly accurate measurements of the forearm position and orientation can be achieved (Passon et al., 2018). In the present work, we build on this previous result and use the improved forearm measurements. However, the methods proposed in Section 3 can likewise be applied when direct measurements of the Diego system or another robotic system are used.

While the Diego system can measure the wrist and elbow position, it has no means for determining the shoulder position. Instead the system requires that the nominal shoulder position ${\;}_{R}^{}{\text{p}}_{\text{S}}^{\text{nom}}$ is defined at the beginning of each trial. For this purpose, the therapist positions the patient into the desired upper-body posture and briefly holds the end of one of the ropes onto the shoulder joint. Throughout the trial, the robotic system estimates the upper arm motion using the assumption that the shoulder remains fixed at the nominal position, as proposed in previous literature (Dipietro et al., 2007; Rosati et al., 2007). This implies that the elbow angle is determined according to (11) but with ${\;}_{R}^{}{\text{x}}_{\text{A}}$ being the normalized vector between the elbow position and the nominal shoulder position. It is assumed that the therapist restraints the trunk or shoulder such that the shoulder position remains constant throughout the session or that the patient follows the instruction to perform the exercise without compensatory motion. In the following, we will compare the results of the *conventional* literature method of the non-augmented system with the results of the proposed *hybrid* method of the inertial-robotic augmented system. We will generally consider the case of a *proper movement* in which the aforementioned assumptions are fulfilled and the case of a *compensatory movement* in which either the fixation is not accomplished correctly or the patient does not follow the instruction, i.e., in both cases the shoulder deviates from its nominal position.

### 4.2. Experimental Setting and Procedure

The proposed methods for hybrid motion tracking and posture biofeedback are evaluated in experiments with five healthy subjects (age of 25–35 years, two female and three male), hereinafter also termed S1–S5. The performed trials involving human participants were reviewed and approved by the ethics committee of the Berlin Chamber of Physicians (Eth-40/15). The chosen subjects cover a large range of body height (160–192 cm) and upper arm length (28–33 cm). During the trials, each subject sits on a chair with the right arm connected to the Diego system as shown in Figure 2. Both ropes of the robotic system Diego are attached at the forearm and the IMU (MTx™, Xsens, Netherlands) is fixed on the lateral aspect of the upper arm (approximately midway along the longitudinal axis). The weight relief of both ropes is adjusted to 6 N to assure tightly stretched ropes, which minimizes position measurement errors. A standard PC (Intel^Ⓡ^,Core™ i5 with four cores) running Linux (Ubuntu 18.04) was utilized to run the software and connect to the devices. The control algorithms and device interfaces were implemented in Matlab/Simulink (MATLAB R2017b; MathWorks, USA) and C/C++ using a modified Linux real-time target to generate an executable (Sojka and Píša, 2014). Using this setup, the methods are found to be highly real-time-capable. The real-time-critical magnetometer-free orientation estimation algorithm part is definitely able to run at the sampling rate of the sensor (here 100 Hz), even on-board the sensor. The most time-consuming part of the methods is the heading estimation including the joint acceleration disagreement. This non-real-time-critical procedure requires about half a second of computation time and must be executed every 5 s in parallel to the real-time-critical part.

**Figure 6:**
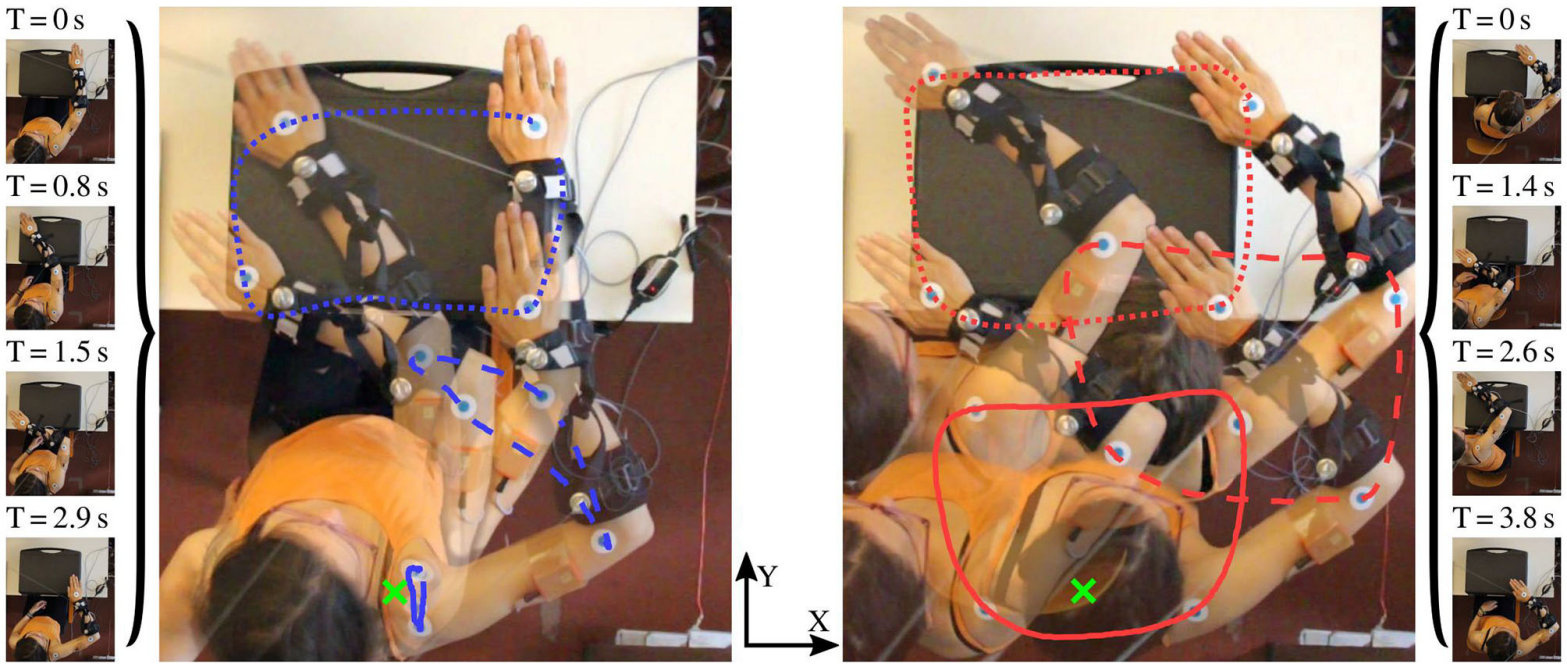
Illustration of the performed motion with and without compensation movements (*comp*.*mov*. and *prop*.*mov*.). The optically tracked paths of the three markers [shoulder (solid), elbow (dashed), hand (dotted)] are highlighted in blue (*prop*.*mov*.) and red (*comp*.*mov*.). The desired (nominal) shoulder position is depicted by the green cross. The time labels above the single frames specify the elapsed time since the beginning of the motion cycle.

A box-shaped object (suitcase) is placed on a table in front of the subject at such height that its top surface is slightly lower than the shoulder. The outer edge of that surface marks a rectangular path that the right hand should follow in counterclockwise cyclic motions, as illustrated in Figure 6. Note that the path is dimensioned and positioned such that the subject can comfortably perform the motion by shoulder joint and elbow joint motions, i.e., without bending the trunk and without considerable displacements of the shoulder. This ensures that each subject can conduct both the proper and the compensatory movements as defined in Section 3.4.

**Figure 7:**
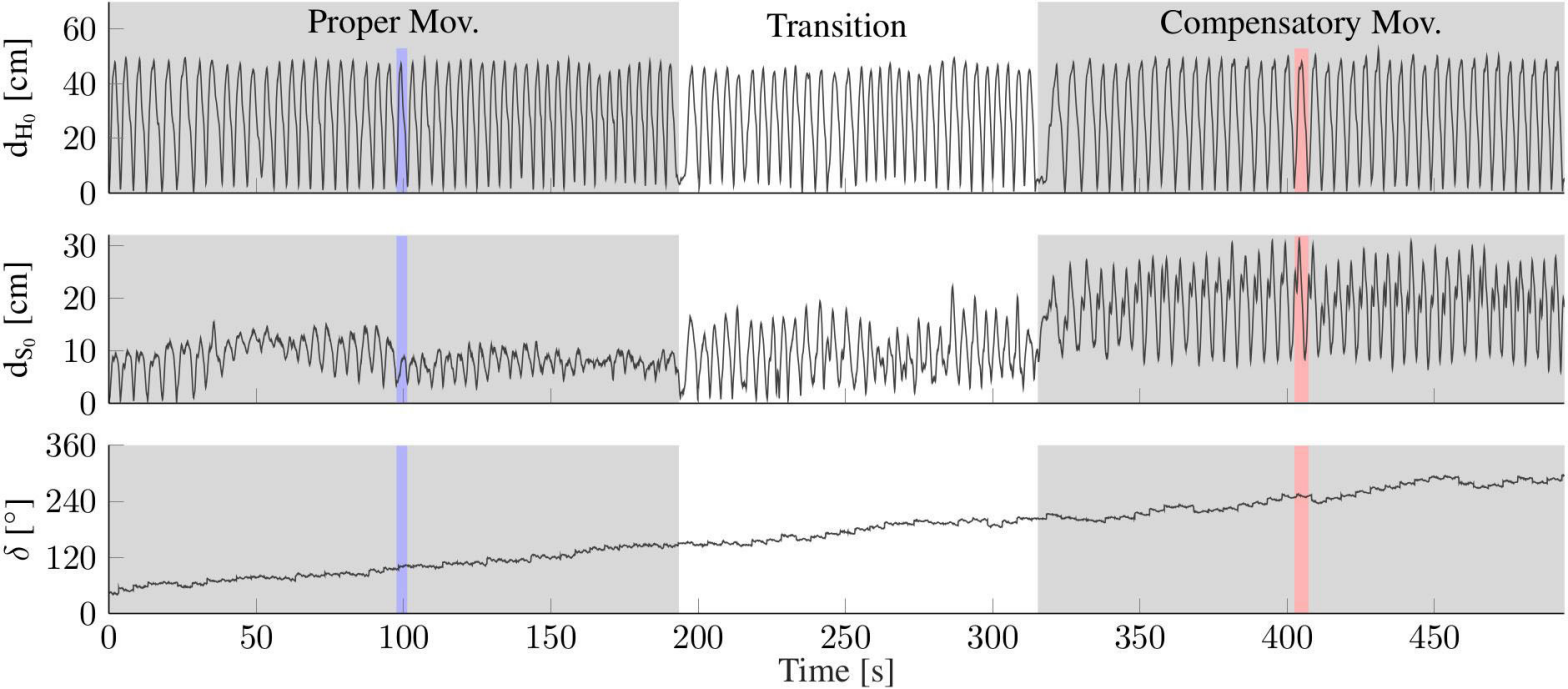
Exemplary data from one complete trial of one subject with colored vertical bands highlighting the time periods of exemplary trials (cf. Figures 8, 9). **(Top/Middle)** Distance of the hand (d_H_0__) and shoulder (d_S_0__) from their respective initial positions at *t* = 0, as measured by the optical reference (*opt*.*ref*.). While hand motion is similar, the proper and compensatory movements exhibit clearly different amounts of shoulder motion. **(Bottom)** The upper arm heading offset (δ) drifts by more than 180° within the 8-min trial.

The subjects are asked to perform each of both movements for time periods of at least two and up to 5 min. In the transition phase between both time periods, the subjects are instructed to slowly increase the level of compensatory movements and to accustom themselves to the unnatural motion performance. Both time periods and the transition phase are indicated in Figure 7, while the difference between proper and compensatory motion is illustrated in Figure 6.

A camera (Canon EOS 600D) is positioned above the subject to obtain reference measurements of positions and angles in the horizontal plane of the motion. The trials are recorded with a frame rate of 50 frames per second and a resolution of 1,280 × 720 pixels. Three adhesive labels (blue filled circle on a bigger white circle, cf. Figure 6) are affixed on the subject's arm—one on the center of the back of the hand, another one on the skin above the center of the shoulder joint, and a third one close to the elbow joint but with sufficient distance to the proximal forearm cuff to assure visibility of the marker from above.

After each trial, the trajectories of the markers are reconstructed from the recorded video by means of the open source software Kinovea (https://www.kinovea.org), and the elbow angle as well as the heading of the upper arm are calculated using standard vector algebra. This yields an approximate ground truth, which is hereafter termed *optical reference* (*opt*.*ref*.).

The robotic workspace coordinate system and the one of the *opt*.*ref*. were initially calibrated and aligned with each other. For this purpose, the length and width of the suitcase were measured. The robot's coordinate system was calibrated by measurements of each cable while they were subsequently pulled to all four edges of the suitcase. For the *opt*.*ref*., the length and width of the suitcase were adjusted in the software Kinovea. The nominal shoulder position ${\;}_{R}^{}{\text{p}}_{\text{S}}^{\text{nom}}$ of the robot alone, as explained in Section 4.1, was initially determined by one rope pulled to the shoulder joint just before each trial. This nominal shoulder position was set as the origin of both the *rob*. and *opt*.*ref*. coordinate systems.

### 4.3. Experimental Results

The proposed method for hybrid motion tracking is validated on recorded data of experimental trials with five subjects as outlined above. On average they performed *prop*.*mov*. for around 4:15 min [3:13 (S2)–5:00 (S4) min] and *comp*.*mov*. for around 2:47 min [2:04 (S4)–4:09 (S1) min]. This resulted in an average of 57 [45 (S2)–69 (S1)] *prop*.*mov*. cycles and in an average of 33 [16 (S4)–48 (S1)] *comp*.*mov*. cycles. The average cycle time of all five subjects is 4.6 s for the *prop*.*mov*. and 5.4 s for the *comp*.*mov*.

**Figure 8:**
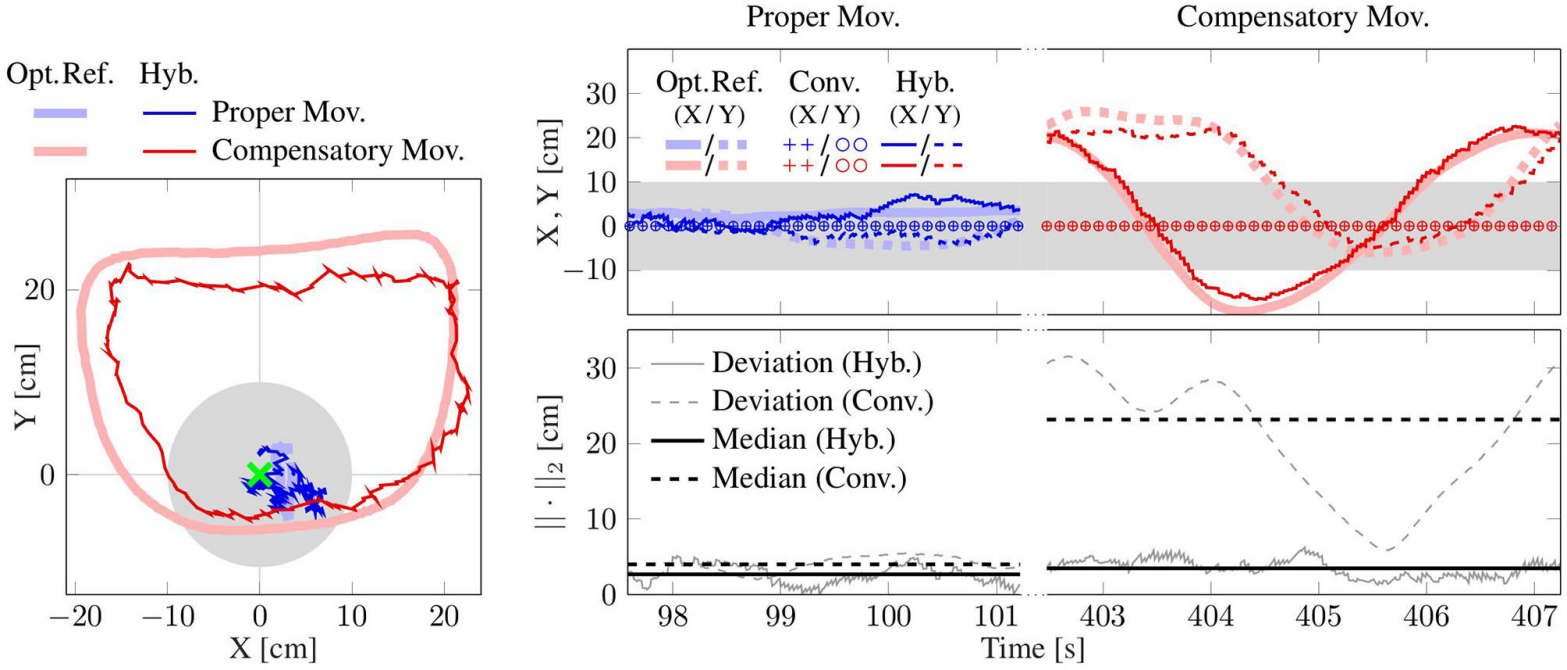
Shoulder motion during a cycle with compensation motion (*Compensatory Mov*.) and a cycle without this undesired motion (*Proper Mov*.); **(Left)**: horizontal-plane trajectories; **(Right)**: x/y-position and measurement error. The proposed method (*Hyb*.) agrees better with the optical reference (*Opt*. *Ref*.) than the conventional method (*Conv*.), which assumes a fixed shoulder. The gray band and circle indicate the shoulder position range that is used for compensatory motion detection.

**Figure 9:**
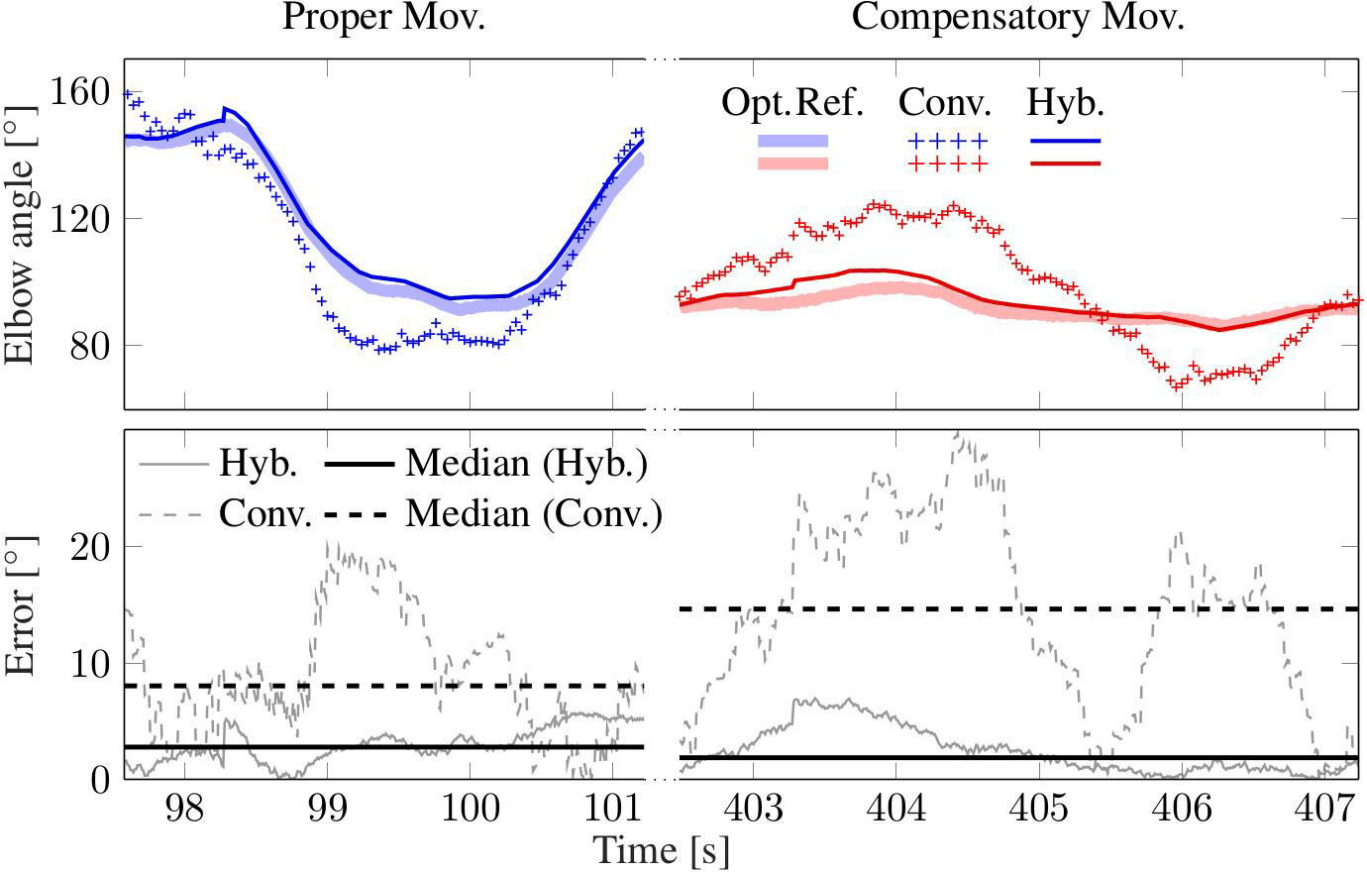
Elbow angle from a trial with compensatory movement (*Compensatory Mov*., red) and another without (*Proper Mov*., blue). The result of the hybrid method (*Hyb*.) agrees well with the optical reference (*Opt*. *Ref*.), while the conventional method (*Conv*.) that assumes a constant shoulder position yields clearly larger errors, especially in the case of *comp*. *mov*.

**Figure 10:**
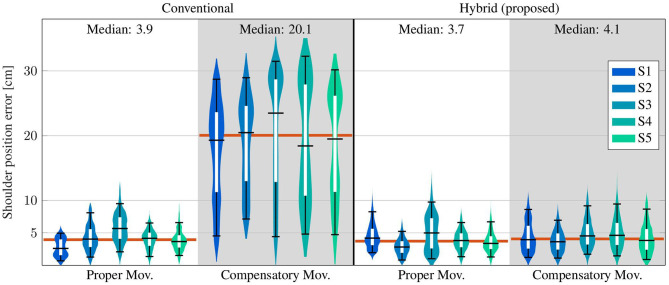
Time-based violin plot of estimated shoulder position deviations (Euclidean distance) from the *opt*. *ref*., with upper/lower whiskers of the inner box plots at the 95th/5th percentile of all values over time. Horizontal red lines indicate median values over all subjects.

**Figure 11:**
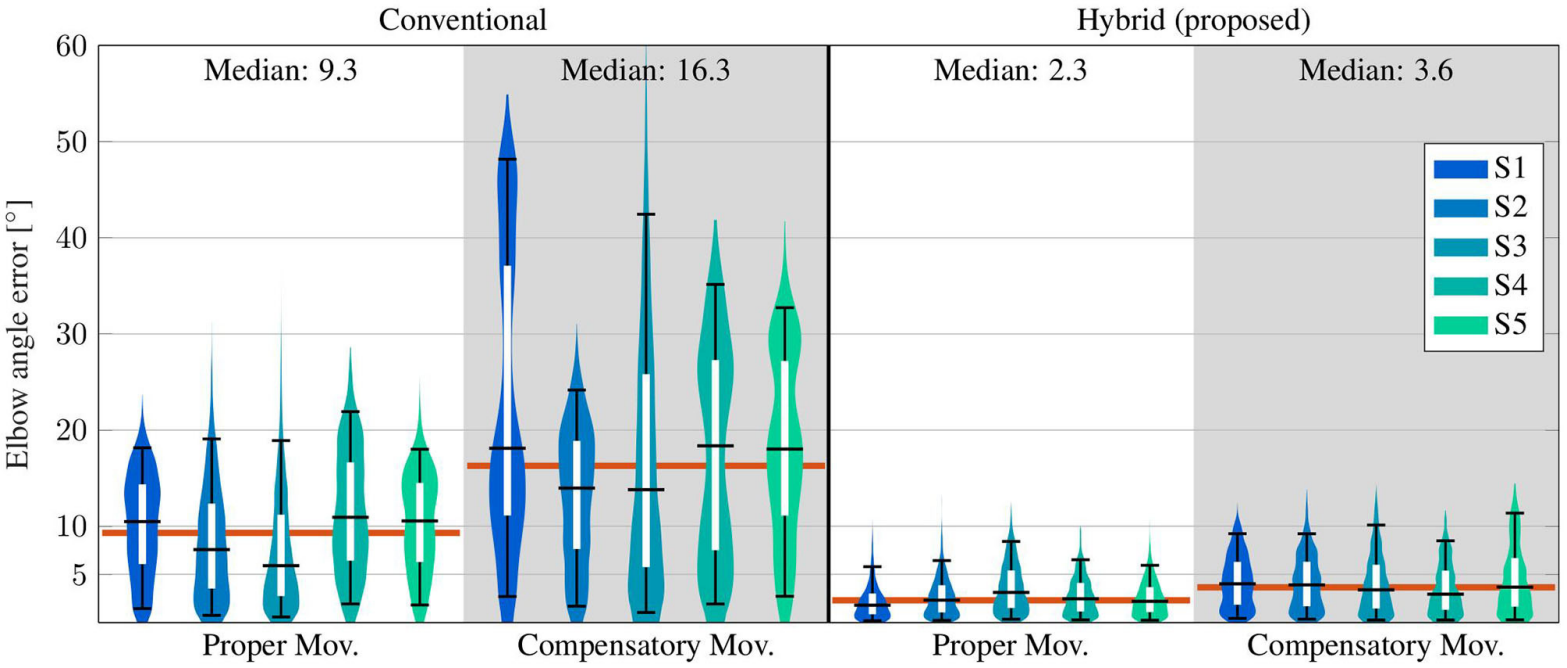
Time-based violin plot of estimated elbow angle deviations from the *opt*. *ref*., with upper/lower whiskers of the inner box plots at the 95th/5th percentile of all values over time. Horizontal red lines indicate median values over all subjects.

**Figure 12:**
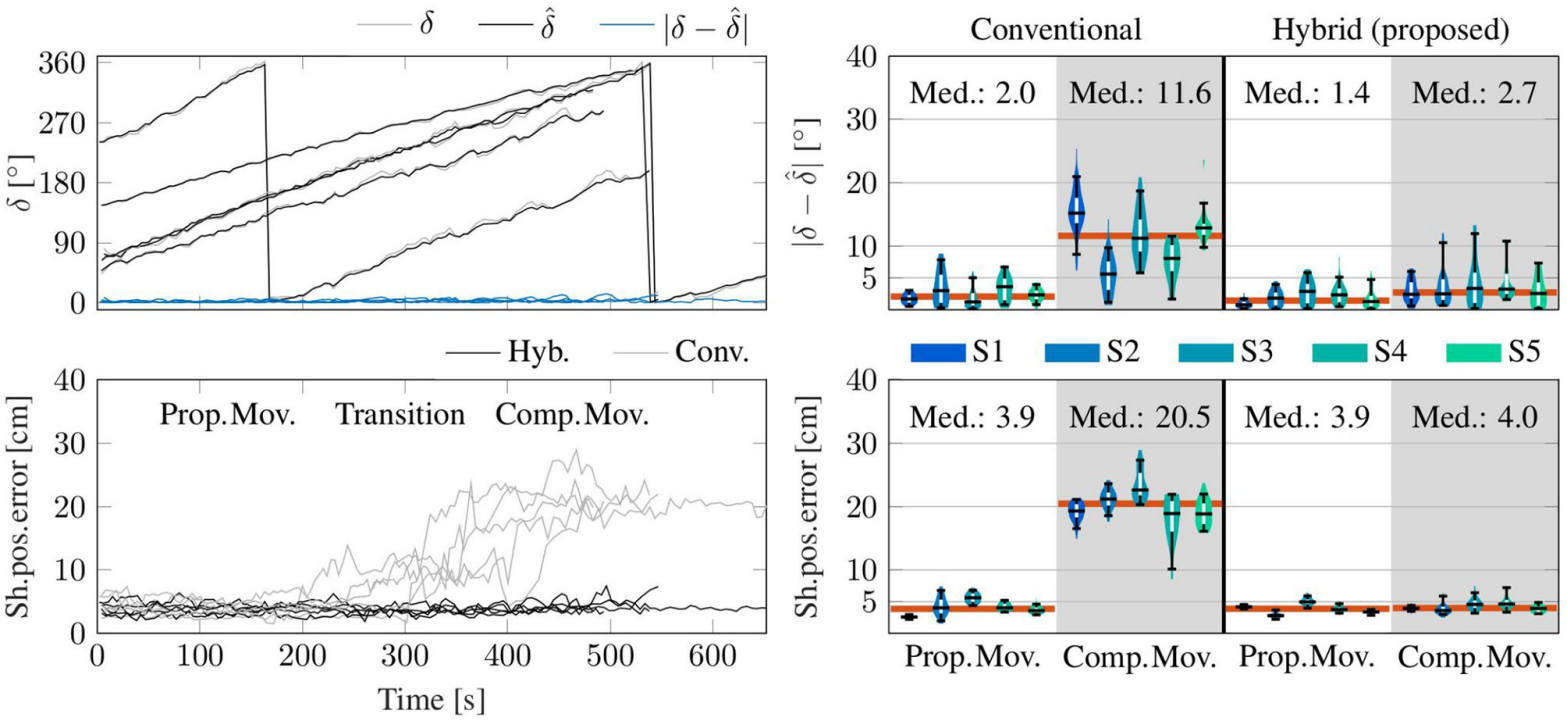
Cycle-based data (medians) from the complete trials of all subjects. **(Top)** The estimated upper arm heading offset ($\hat{\delta }$) agrees well with the true offset δ between the upper arm headings of the optical reference and of the uncorrected orientation estimate by the IMU (all as medians of each trial iteration). **(Bottom)** Errors of the shoulder position with respect to the *opt*. *ref*. as medians of each trial iteration. In the clear majority (91 %) of all trials of all subjects, the median error of the proposed method is smaller than five centimeters, while the conventional method (*Conv*.) fails to determine adequate shoulder positions during *comp*. *mov*. and in the transition phase. **(Right)** Corresponding violin plots of the plots on the left excluding the transition phase. Upper/lower whiskers of the inner box plots show the 95th/5th percentiles. Horizontal red lines indicate median values over all subjects.

The validation results of all five subjects are given in Figures 10–12 as well as in Tables 1–3. Detailed insights are provided by plots of exemplary data from S2 shown in Figures 7–9 as well as in Figure 6.

**Table 1:** Estimated shoulder position deviations (Euclidean distance) from *opt*.*ref*., medians over all subjects in centimeter.

**Type**	**Method**	**Median**	**Probability/Correlation**
Proper mov.	Conv.	3.9	}*p* = 0.106, *r* = 0.003
	Hyb.	3.7	
Compensatory mov.	Conv.	20.1	}*p* < 0.001, *r* = 0.845
	Hyb.	4.1	

*A Welch's T-test (probability p and Pearson correlation coefficient r) between Conv. and Hyb. yields no significant effect for prop.mov., but a large effect for comp.mov*.

**Table 2:** Estimated elbow angle errors with respect to *opt*.*ref*., median values over all subjects in degree.

**Type**	**Method**	**Median**	**Probability/Correlation**
Proper mov.	Conv.	9.3	}*p* < 0.001, *r* = 0.706
	Hyb.	2.3	
Compensatory mov.	Conv.	16.3	}*p* < 0.001, *r* = 0.724
	Hyb.	3.6	

*A Welch's T-test (probability p and Pearson correlation coefficient r) between Conv. and Hyb. yields large effects for both, prop. and comp. mov*.

**Table 3:** Table of confusion for real-time detection of compensatory motion from data of a single motion cycle.

		**Predicted type**	
**Subject**	**Actual type**	**Prop**.	**Comp**.
S1	Prop.	69	0
	Comp.	0	48
S2	Prop.	45	0
	Comp.	0	40
S3	Prop.	61	0
	Comp.	0	31
S4	Prop.	47	0
	Comp.	0	16
S5	Prop.	62	0
	Comp.	0	28

Figure 7 presents the motion of the hand and shoulder with respect to the initial position that is defined at time 0. The figure also depicts the heading offset δ for the entire duration of the experiment for S2. The hand moved along the same path throughout the experiment. However, while the shoulder displacements are mostly below 10 cm for *prop*. *mov*., the shoulder deviates between 10 and 30 cm during the *comp*. *mov*. from the nominal shoulder position. The colored vertical bands in Figure 7 highlight the time periods of exemplary trials, for which detailed data is presented in Figures 8, 9. In Figure 6, for both highlighted time periods, four still images of the camera system are superimposed, and trajectories of the hand, elbow and shoulder markers are indicated.

The plot at the bottom of Figure 7 shows that the heading offset of the magnetometer-free inertial orientation estimation is drifting at ~0.5 deg/s during the entire experiment due to integration of gyroscope bias. This observation is in good agreement with *a-posteriori* analysis of the gyroscope readings during rest, which revealed bias magnitudes of 0.1 – 0.76 deg/s (average of 0.34 deg/s) for the utilized IMU. Note that the proposed method does *not* require initial rest phases or static gyroscope calibration and that the aforementioned bias values were only determined for validation purposes but *not* removed from the gyroscope readings at any point.

#### 4.3.1. Shoulder Position Results

The shoulder position is estimated using the upper arm orientation and the elbow position (see Section 3.4). For one exemplary trial of both movement types, Figure 8 depicts the motion in the horizontal plane. The deviation from the *opt*. *ref*. is calculated using the Euclidean norm. The *hyb*. estimates shown in Figure 8 agree well with the *opt*. *ref*., with median values below 4 cm. The *conv*. *method* assumes a fixed shoulder position, which works adequately for *prop*. *mov*. but yields errors above 20 cm during the depicted *comp*. *mov*. cycle. Here, the robot does not measure any shoulder motion and the resulting *conv*. errors reproduce the actually conducted shoulder motion as measured by the *opt*. *ref*. The presented exemplary results are consistent with the medians over all subjects (see Table 1), which are all close to 4 cm except for the median of 20.1 cm of the *conv*. *method* during *comp*. *mov*.

Figure 10 shows the distributions of the deviations from the *opt*. *ref*. for conventional (*conv*.) measurements and for the results of the proposed hybrid (*hyb*.) methods. The upper whisker represent the 95th percentile and the lower one the 5th percentile of all values over time. The inner boxes themselves depict the quartiles, i.e., the 25th percentile, the median and the 75th percentile. All time-based medians of the proposed *hyb*. *method* are near or below 5 cm, and all corresponding upper whiskers stay below 10 cm. The results of the conventional (*conv*.) method are comparable for the *prop*. *mov*., but reach significantly higher time-based medians of up to 23.5 cm (S3) and upper-whisker values up to 32.3 cm (S4) for the *comp*. *mov*. Welch's *T*-test states a large effect for *comp*. *mov*. (probability *p* < 0.001 and Pearson correlation coefficient *r* = 0.845). No significant effect is stated for *prop*. *mov*. with *p* = 0.106 and *r* = 0.003.

#### 4.3.2. Elbow Angle Results

The elbow angles of exemplary motion cycles are shown in Figure 9. The results, as presented in that figure, of the proposed *hyb*. *method* resemble the *opt*. *ref*. signals even during *comp*. *mov*., and medians below 3° are achieved. In contrast, deviations of up to 20° occur with the *conv*. *method* even in the *prop*. *mov*. cycle, and the median during the *comp*. *mov*. cycle reaches almost 15°. These results are consistent with the results over all subjects, as presented in Table 2 and Figure 11. All time-based median deviations between the *hyb*. measurement and the *opt*. *ref*. are near or below 4°, and the 95th percentiles mostly stay below 10°. The *conv*. measurements yield significantly larger time-based medians in the range of 5.9–18.4° and upper whiskers with up to 48.2°. Welch's *T*-test states large effects for both, *prop*. and *comp*. *mov*., with *p* < 0.001 and *r* > 0.7.

#### 4.3.3. Parameter Sensitivity Analysis

The distance ${\;}_{A}^{}{\text{p}}_{\text{E}}$ of the IMU to the elbow joint, the length *l*_A_ of the adjacent segment (here upper arm) and the cutoff frequency for the determination of the time derivative ${\;}_{A}^{}{\overset{∙}{\omega }}_{\text{A}}$ have to be manually determined or chosen. We perform a parameter sensitivity analysis to investigate their impact on the estimated shoulder position and elbow angle. None of these parameters is particularly sensitive.

The distance ${\;}_{A}^{}{\text{p}}_{\text{E}}$ was chosen as 15 cm, which is approximately half the average upper arm length of humans; e.g., Chaffin et al. (2006) recommend 28.1 and 29.8 cm for the upper arm length of female and male humans, respectively. We now consider the case in which the assumed and the actual IMU-to-elbow distance differ by 7.5 cm, and we investigate how the measurement errors reported in Sections 4.3.1 and 4.3.2 are affected by this parameter change. The median measurement deviations increase by 0.06 cm (shoulder position) and 0.21° (elbow angle) on average over all subjects. This corresponds to relative changes of <2 % in the median shoulder position error and <8 % in the median elbow angle error. The 95th percentile errors increase by 0.17 cm and 0.41° on average over all subjects, respectively. This corresponds to relative changes of <3 % in the 95th percentile shoulder position error and <6 % in the 95th percentile elbow angle error. The corresponding intra-subject changes are reported in detail in Supplementary Tables 1, 2.

The length *l*_A_ of the upper arm was measured manually. We now consider the case in which the measured upper length differ by ±2.5 cm, and we investigate how the measurement errors are affected by these parameter variations. They have no impact on the accuracy of the elbow angle results but on the shoulder position error. We call that this error is defined as the difference between the *hybrid* and the *opt*. *ref*. displacements of the shoulder with respect to the nominal position. Since the considered parameter change also affects the nominal shoulder position of the *hybrid* measurement, it does not lead to a direct offset in the shoulder displacement but has a more indirect effect. The median measurement errors of the shoulder position change by 0.05 cm (*l*_A_ + 2.5 cm) and 0.07 cm (*l*_A_ − 2.5 cm) on average over all subjects. This corresponds to relative changes of <2 % in the median shoulder position error. The 95th percentile errors change by 0.58 and −0.36 cm on average over all subjects, respectively. This corresponds to relative changes of <8 % in the 95th percentile shoulder position errors. The corresponding intra-subject changes are reported in detail in Supplementary Table 3.

The cutoff frequency for the determination of the time derivative ${\;}_{A}^{}{\overset{∙}{\omega }}_{\text{A}}$ was chosen as 2.5 Hz. We now consider variations of ±1 Hz, and we investigate how the measurement errors are affected by these parameter changes. The median measurement deviations change by <0.005 cm (cutoff frequency + 1 Hz) and by −0.01 cm (cutoff frequency − 1 Hz) for the shoulder position, and by <0.005° (cutoff frequency ± 1 Hz) for the elbow angle on average over all subjects. This corresponds to relative changes of <1 % in the median shoulder position error and <1 % in the median elbow angle error. The 95th percentile errors change by <0.005 cm and <0.005° (cutoff frequency ± 1 Hz) on average over all subjects, respectively. This corresponds to relative changes of <1 % in the 95th percentile shoulder position errors and <1 % in the 95th percentile elbow angle errors. The corresponding intra-subject changes are reported in detail in Supplementary Tables 4, 5.

#### 4.3.4. Long-Time Stability Analysis

The cycle-based medians of the shoulder position errors of the hybrid method and the conventional method with respect to the *opt*. *ref*. are shown in Figure 12. It is evident that the results of the proposed *hyb*. *method* constantly remain around 3–5 cm and no linear long-time trend is present. This is in line with the results presented in the violin plot on the bottom right of Figure 12: the median cycle-based errors between the *hyb*. measurement and the *opt*. *ref*. stay below 5 cm for all subjects (95th percentiles between 3.7 and 7.4 cm) and around 4 cm over all subjects. The *conv*. measurements yield significantly larger medians up to 22.6 cm (20.5 cm over all subjects) and upper whiskers with up to 27.3 cm in the case of *comp*. *mov*.. The estimated upper arm heading offset $\hat{\delta }$ is also depicted as cycle-based medians over all trials of all subjects and reveals long-time stable estimates of the heading offset with absolute errors remaining under 5° for 90 % of the measurements. This is in line with the results shown in the violin plot on the top right of Figure 12: the median absolute errors of the estimated upper arm heading offset stay below 4° for all subjects (95th percentiles between 1.7 and 12.0°) and the median absolute errors over all subjects are 1.4 and 2.7° for *prop*. *mov*. and *comp*. *mov*., respectively.

#### 4.3.5. Compensatory Motion Detection Results

The estimated shoulder position is utilized to detect compensatory movements, for which biofeedback can be provided as described in Section 3.4. A tolerated compensatory shoulder motion range of ±10 cm is shown in the exemplary plots of Figure 8. The detection algorithm identifies *comp*. *mov*., i.e., displacements of the shoulder which might be due to a not correctly fixated patient's shoulder or that the patient does not follow the instruction to move with a fixed shoulder position, when the tolerated shoulder range of ±10 cm is exceeded for more than 20 % of the duration of a trial. Table 3 demonstrates that all 284 *prop*. *mov*. cycles are correctly recognized as proper and that all 163 *comp*. *mov*. cycles are likewise correctly classified, i.e., all 447 cycles out of 447 cycles are correctly classified.

## 5. Discussion

The proposed augmentation of end-effector-based robotic systems leads to considerable improvements in the considered application scenario of the cable-based upper-limb rehabilitation robot. It provides accurate shoulder motion measurements in real time with time-based median measurement errors around 4 cm (all 95th percentiles below 10 cm) as well as real-time elbow angle measurements with time-based median errors below 4° (95th percentiles mostly below 10°). This is, particularly during compensatory movements, a significant improvement compared to the conventional approach of assuming a fixed shoulder position during end-effector-based therapy (Dipietro et al., 2007; Rosati et al., 2007). In Section 4.3.4, it is shown that the cycle-based medians of the shoulder position errors of the proposed method constantly remain around 3–5 cm (91 % are smaller than 5 cm). Thus, the accuracy over one course of the conducted motion is within the range, which meets the requirements we defined toward the end of Section 2.

This accuracy is comparable to the results presented by Bertomeu-Motos et al. (2015a), which uses a magnetometer-based approach that was shown to depend on unrealistic or restrictive homogeneity properties of the magnetic field (Madgwick et al., 2011; Seel and Ruppin, 2017; Salchow-Hömmen et al., 2019). Such an assumption is known to be violated if robot components, nearby furniture or objects that are handled by the subjects contain iron or other ferromagnetic materials or electronic components. In fact, magnetic fields inside buildings are so inhomogeneous that their patterns can be analyzed for indoor localization and mapping (Le Grand and Thrun, 2012; Subbu et al., 2013; Shu et al., 2015). For this reason magnetometers do *not* provide reliable heading information for robust motion tracking in indoor environments. The fact that the methods proposed in Section 3 are magnetometer-free makes them highly suitable for indoor applications and realistic robotic environments. Long-time stability of the estimated positions and orientations is achieved, and the proposed methods are highly real-time-capable as described above. The combination of these properties defines the novelty of the current approach with respect to previous methods. One previous method (Wittmann et al., 2019) exploits the patterns of the magnetic field in indoor environments and only relies on IMUs. It provides accurate estimates of the arm motion, however it requires the user to rest in-between the therapy session to re-correct the magnetometer-based drift. In contrast, the proposed method is magnetometer-free and does not need that the user rests at any time. Additionally, applied rehabilitation robots modify the magnetic field due to their ferromagnetic materials, which will influence the accuracy of the method by Wittmann et al. (2019). Furthermore, the proposed approach provides the shoulder joint position in the robotic coordinate system, which cannot be provided by algorithms that do not include measurements of the robot. This is also the case for other methods providing accurate estimates of the arm orientation solely based on IMUs (Kok et al., 2014). The latter approach by Kok et al. (2014) is even magnetometer-free, however it cannot directly be implemented in real-time. The proposed method of the current article is novel in the sense that it provides long-time stable, magnetometer-free, and real-time estimates of the orientation of the adjacent segment and the endpoint of that segment, e.g., shoulder position, in the robotic coordinate frame.

Although all motions are performed in a horizontal plane, the two-dimensional optical motion tracking yields only an approximate ground truth. In preliminary trials, we investigated the variance of the distance between the elbow and shoulder marker. This distance, which should ideally be constant, has been found to have standard deviations between at least 0.7 cm (S2) and at most 1.2 cm (S3) around their mean value. We conclude that this approximate ground truth is sufficiently precise for the desired proof of concept.

Limitations of the validation are that healthy subjects performed the motions and that the accuracy of the approximate optical ground truth does not achieve the same level as the golden standard of marker-based stereophotogrammetric tracking systems, which is mainly due to the horizontal projection, distortions by the camera lens symmetry, and marker displacement caused by skin and muscle motion. As mentioned above, the resulting inaccuracies of the camera-based reference measurements are below 5 cm, which is small enough for the present proof-of-concept study but not small enough to decide whether the proposed method yields accuracies below state-of-the-art results. Furthermore, a limitation of the proposed methods is the number of parameters and that their ideal choice is not yet clear. However, first investigations, as described in Section 4.3.3, revealed that their sensitivity against changes is not severe. Variations of the cutoff frequency of ±1 Hz cause only negligible changes in the range of 1% in the measurement accuracy. The sensitivity of the measurement errors to displacements of the IMU seems acceptable in practice if the user places the sensor within a 15 cm (±7.5 cm) wide area around the middle of the upper arm; even a distance of 7.5 cm between assumed and actual position causes error increases below 8% of the original error. To interpret the sensitivity of the proposed methods against inexact upper-arm lengths, consider a 1.50 and a 1.90 m tall subject with average upper-arm lengths of 27.9 and 35.3 cm, respectively Winter (2005). Even if one simply uses the proposed 30 cm for both subjects, the measurement error increase can be expected to stay below 5% in both cases. In sum, the proposed methods are not very sensitive against these parameters and they can be used with the proposed values in practice without jeopardizing the measurement accuracy.

It is worth noting that the proposed methods can be extended to cases in which the motion of more segments than only the directly adjacent body segment is of interest. For example, if the foot is connected to an end-effector-based robotic system, then we might want to track the motion of the shin and the thigh. In such a case, the proposed method can be used with an IMU on the shin to track the knee position. This knee position estimate might then be used to apply the method again with an IMU on the thigh and determine the hip joint position. While the feasibility of such a cascaded approach follows directly from the properties of the proposed methods, further research is needed to investigate which levels of accuracy can be achieved in practice and also which performance is achieved by the proposed methods in other application scenarios.

As mentioned in Section 1, preventing compensation during rehabilitation training improves the therapy outcome and decreases long-term problems, such as pain, orthopedic illnesses and learned non-use (Levin et al., 2009). One possibility to avoid compensation are trunk and shoulder girdle restraints. Their effects are discussed diversely in the literature. For example, 5 of 8 studies named by Greisberger et al. (2016) showed improvements of arm motion recovery, whereas one of the included studies states auditory feedback as more effective on movement patterns directly after training, and the other two studies did not reveal any effect. In conclusion, Greisberger et al. (2016) considered the magnitude of change of the observed improvements as not consistently clinically relevant. Indisputably there is an additional donning and doffing effort, as well as a restriction of natural trunk and shoulder girdle motion, which can also be seen in healthy people performing arm movements. These disadvantages can be avoided by using real-time biofeedback. In contrast to trunk and shoulder restraint, biofeedback can be adapted to the needs of the individual, e.g., in the case of progress (Valdés and der Loos, 2017). Furthermore, indications suggest that improvements are rather maintained after feedback that is only provided when it is needed than after training with concurrent physical guidance (Schmidt and Lee, 2014). One possibility to detect compensation motion would be the additional use of more IMUs as, e.g., on the torso. However, the tracking of the trunk or shoulder position is severely limited due to the occurring position drift. It is, of course, possible to detect inclination changes, but translational motion of the trunk without bending the torso would not be detectable nor drift-free estimable. To detect motions of the shoulder girdle, even more IMUs and thus more donning and doffing would be required. Using only one IMU and the already available measurements of the robot, as proposed here, reduces the hardware effort and provides a drift-free and accurate tracking of the shoulder position, that can be utilized to detect compensation motion of the trunk and shoulder. In sum, the natural trunk and shoulder motion is not restricted with the proposed solution, while still the full monitoring of compensatory movements is possible facilitating biofeedback when needed. Cirstea and Levin (2000) found mean shoulder displacements of 14 cm for moderate to severe impaired stroke subjects and around 4 cm for healthy participants. Valdés and der Loos (2017) stated 3 cm of shoulder-spine motion as physiological movements. The experimental results in Section 4.3 show that the proposed method can reliably distinguish between small shoulder displacements and shoulder displacements of more than 10 cm that are performed to compensate reduced motion in other joints. The proposed and applied toleration of range violations for up to 20% of the trial duration can of course be replaced by a tolerated time period in case of non-repetitive motions. In our case, the 20% of the trial duration equated to a time period of one second, which would have leaded to the same perfect classification results. It is, of course, necessary to determine the appropriate degree of compensatory motion detection in clinical use, individually for the desired therapy application and setting, i.e., when is a biofeedback helpful and not useless or annoying.

## 6. Conclusions

End-effector-based rehabilitation robots offer motion support with fast and easy robot-to-patient setup and adjustment. This advantage is, however, achieved at the cost of a reduced amount and accuracy of motion measurement information. A conventional solution is to rely on a fixed shoulder position assumption (Dipietro et al., 2007; Rosati et al., 2007). We demonstrated that these limitations can be overcome by a hybrid system design that uses wearable inertial sensors and real-time sensor fusion methods without requiring a clear line-of-sight and thus overcomes a major restriction in depth-camera-based designs. The proposed approach accurately tracks the motion of a body segment that is adjacent to the robot-connected body segment. It assures long-time stability and complete immunity to magnetic disturbances, which are common in indoor applications and robotic environments. The generalizability and the transfer of the proposed method's benefits to other kinematic chains and application scenarios are expected but cannot be guaranteed from the investigated particular setup. Consequently, this has to be investigated and demonstrated in future work.

We demonstrated that the method can be used to infer real-time estimates of the complete orientation of the upper arm and the shoulder position in the robotic frame. This enables the detection of undesirable compensatory trunk and shoulder motions in upper-limb rehabilitation training and thus facilitates real-time biofeedback, which is expected to improve active involvement and therapy outcome (Levin et al., 2009). Clinical validation in neurological patients will be subject of future research. Simultaneously, it will be investigated if the hybrid system can be utilized for feedback-controlled Functional Electrical Stimulation (FES) and what its impact on such solutions is.

Beyond the proposed methods, we believe that the general approach of augmenting easy to setup end-effector-based robotic systems with wearable sensors is promising and might provide additional advantages over existing solutions in a range of application scenarios, in which accurate real-time motion tracking is required to realize feedback control, objective motion assessment or biofeedback.

## Data Availability Statement

The raw data supporting the conclusions of this article will be made available by the authors, without undue reservation.

## Ethics Statement

The studies involving human participants were reviewed and approved by Berlin Chamber of Physicians. The patients/participants provided their written informed consent to participate in this study. Written informed consent was obtained from the individual(s) for the publication of any potentially identifiable images or data included in this article.

## Author Contributions

AP, TSc, and TSe: conceptualization, investigation, and writing—review and editing. AP and TSe: formal analysis, methodology, software, visualization, and writing—original draft. TSc and TSe: funding acquisition and supervision. All authors contributed to the article and approved the submitted version.

## Conflict of Interest

The authors declare that the research was conducted in the absence of any commercial or financial relationships that could be construed as a potential conflict of interest.
