# Supplementary material for: Inertial-Robotic Motion Tracking in End-Effector-Based Rehabilitation Robots
Source: Front Robot AI. 2020 Nov 27;7:554639. doi: 10.3389/frobt.2020.554639 (PMC7806092; doi:10.3389/frobt.2020.554639)
Supplement: Supplementary file 1 [file Data_Sheet_1.PDF]

frontiers

Supplementary Material

Inertial-Robotic Motion Tracking in End-Effector-based Rehabilitation Robots

Table S1. Parameter sensitivity of the estimated shoulder position errors (Euclidean distance) for a large disagreement between the assumed and the actual IMU-to-elbow distance ${}_{\mathcal{A}}\mathbf{p}_{\mathrm{E}}$. Abbreviations: Proper movement (Prop.Mov.), Compensatory movement (Comp.Mov.), Distance (Dist.), Percentile (%ile).

Subject	Type	Original Error [cm] Dist. IMU to Elbow ${}_{\mathcal{A}}\mathbf{p}_{\mathrm{E}}$ = 15 cm			Change of the Errors [cm] Changed to half value: ${}_{\mathcal{A}}\mathbf{p}_{\mathrm{E}}$ = 7.5 cm		
		Median	5th %ile	95th %ile	Median	5th %ile	95th %ile
S1	Prop.Mov.	4.19	1.94	8.25	0.12	0.01	0.17
	Comp.Mov.	3.94	1.21	8.61	0.06	0.09	-0.09
S2	Prop.Mov.	2.81	0.80	5.23	0.09	0.00	0.14
	Comp.Mov.	3.63	1.11	6.95	0.06	-0.03	0.20
S3	Prop.Mov.	4.98	1.00	9.75	0.14	0.06	0.31
	Comp.Mov.	4.52	1.70	9.17	-0.10	-0.19	0.24
S4	Prop.Mov.	3.79	1.35	6.59	0.10	0.00	0.11
	Comp.Mov.	4.60	1.43	9.40	-0.03	-0.08	-0.14
S5	Prop.Mov.	3.37	1.28	6.70	0.04	-0.01	0.13
	Comp.Mov.	3.83	0.88	8.66	0.11	0.11	0.65
	**Average**	**3.97**	**1.27**	**7.93**	**0.06**	**0.00**	**0.17**
		**Average relative change**			**1.5 %**	**-0.3 %**	**2.2 %**

Table S2. Parameter sensitivity of the estimated elbow angle errors for a large disagreement between the assumed and the actual IMU-to-elbow distance ${}_{\mathcal{A}}\mathbf{p}_{\mathrm{E}}$. Abbreviations: Proper movement (Prop.Mov.), Compensatory movement (Comp.Mov.), Distance (Dist.), Percentile (%ile).

Subject	Type	Original Error [°] Dist. IMU to Elbow ${}_{\mathcal{A}}\mathbf{p}_{\mathrm{E}} = 15\,\mathrm{cm}$			Change of the Errors [°] Changed to half value: ${}_{\mathcal{A}}\mathbf{p}_{\mathrm{E}} = 7.5\,\mathrm{cm}$		
		Median	5th %ile	95th %ile	Median	5th %ile	95th %ile
S1	Prop.Mov.	1.79	0.17	5.79	0.05	0.00	-0.01
	Comp.Mov.	4.01	0.41	9.23	-0.28	-0.01	-0.47
S2	Prop.Mov.	2.31	0.21	6.43	0.13	0.00	0.12
	Comp.Mov.	3.90	0.34	9.22	0.19	0.03	0.62
S3	Prop.Mov.	3.12	0.33	8.42	0.38	0.02	1.04
	Comp.Mov.	3.38	0.24	10.13	0.00	0.12	-0.75
S4	Prop.Mov.	2.50	0.27	6.53	0.25	0.02	0.95
	Comp.Mov.	2.95	0.24	8.41	0.14	0.12	0.24
S5	Prop.Mov.	2.19	0.22	5.94	0.13	-0.01	0.37
	Comp.Mov.	3.67	0.27	11.37	1.14	0.37	1.94
	**Average**	**2.98**	**0.27**	**8.15**	**0.21**	**0.07**	**0.41**
			**Average relative change**		**7.1 %**	**24.4 %**	**5.0 %**

Table S3. Parameter sensitivity of the estimated shoulder position errors (Euclidean distance) for typical to-be-expected variations ($\pm$2.5 cm) in the manually measured upper arm length l_A. Abbreviations: Proper movement (Prop.Mov.), Compensatory movement (Comp.Mov.), Percentile (%ile).

Subject	Type	Original Error [cm]			Change of the Errors [cm]					
		Upper arm length l_A: measured			Changed: l_A +2.5 cm			Changed: l_A -2.5 cm		
		Median	5th %ile	95th %ile	Median	5th %ile	95th %ile	Median	5th %ile	95th %ile
S1	Prop.Mov.	4.19	1.94	8.25	-0.01	0.04	0.75	0.18	-0.13	-0.61
	Comp.Mov.	3.94	1.21	8.61	-0.04	-0.26	0.85	0.19	0.35	-0.65
S2	Prop.Mov.	2.81	0.80	5.23	0.06	-0.07	0.35	0.07	0.09	0.17
	Comp.Mov.	3.63	1.11	6.95	-0.01	-0.06	0.13	0.02	0.11	-0.06
S3	Prop.Mov.	4.98	1.00	9.75	0.38	0.10	0.95	-0.21	-0.03	-0.72
	Comp.Mov.	4.52	1.70	9.17	-0.07	-0.46	0.78	0.18	0.54	-0.46
S4	Prop.Mov.	3.79	1.35	6.59	0.15	-0.06	0.60	-0.05	0.23	-0.21
	Comp.Mov.	4.60	1.43	9.40	-0.01	-0.07	-0.12	0.10	0.20	0.17
S5	Prop.Mov.	3.37	1.28	6.70	0.12	-0.13	0.77	0.05	0.13	-0.60
	Comp.Mov.	3.83	0.88	8.66	-0.05	0.15	0.72	0.15	0.22	-0.65
**Average**		**3.97**	**1.27**	**7.93**	**0.05**	**-0.08**	**0.58**	**0.07**	**0.17**	**-0.36**
		**Average relative change**			**1.3%**	**-6.5%**	**7.3%**	**1.7%**	**13.5%**	**-4.6%**

Table S4. Parameter sensitivity of the estimated shoulder position errors (Euclidean distance) for variations of the cutoff frequency of the Butterworth filter for the determination of the time derivative ${}_{\mathcal{A}}\dot{\omega}_{\mathrm{A}}$. Abbreviations: Proper movement (Prop.Mov.), Compensatory movement (Comp.Mov.), Percentile (%ile).

Subject	Type	Original Error [cm] Cutoff frequency = 2.5 Hz			Change of the Errors [cm] Changed: Cutoff frequency +1 Hz			Changed: Cutoff frequency -1 Hz		
		Median	5th %ile	95th %ile	Median	5th %ile	95th %ile	Median	5th %ile	95th %ile
S1	Prop.Mov.	4.19	1.94	8.25	0.00	0.00	0.00	-0.01	0.00	0.00
	Comp.Mov.	3.94	1.21	8.61	0.00	0.00	0.00	0.00	0.00	0.01
S2	Prop.Mov.	2.81	0.80	5.23	0.00	0.00	0.00	0.00	0.00	0.00
	Comp.Mov.	3.63	1.11	6.95	0.00	0.00	0.00	0.00	0.00	0.00
S3	Prop.Mov.	4.98	1.00	9.75	0.01	-0.01	0.01	0.00	0.00	0.00
	Comp.Mov.	4.52	1.70	9.17	0.00	0.00	0.01	0.00	0.00	0.00
S4	Prop.Mov.	3.79	1.35	6.59	0.00	0.00	0.00	0.00	0.00	0.00
	Comp.Mov.	4.60	1.43	9.40	0.00	0.00	0.00	0.00	0.00	0.00
S5	Prop.Mov.	3.37	1.28	6.70	0.00	0.00	0.00	0.00	0.00	0.00
	Comp.Mov.	3.83	0.88	8.66	0.00	0.00	0.00	-0.08	0.00	0.00
**Average**		**3.97**	**1.27**	**7.93**	**0.00**	**0.00**	**0.00**	**-0.01**	**0.00**	**0.00**
		**Average relative change**			**0.0 %**	**-0.1 %**	**0.0 %**	**-0.2 %**	**0.0 %**	**0.0 %**

Table S5. Parameter sensitivity of the estimated elbow angle errors for variations of the cutoff frequency of the Butterworth filter for the determination of the time derivative ${}_{\mathcal{A}}\dot{\boldsymbol{\omega}}_{\mathrm{A}}$. Abbreviations: Proper movement (Prop.Mov.), Compensatory movement (Comp.Mov.), Percentile (%ile).

Subject	Type	Original Error [°]			Change of the Errors [°]					
		Cutoff frequency = 2.5 Hz			Changed: Cutoff frequency +1 Hz			Changed: Cutoff frequency -1 Hz		
		Median	5th %ile	95th %ile	Median	5th %ile	95th %ile	Median	5th %ile	95th %ile
S1	Prop.Mov.	1.79	0.17	5.79	0.00	0.00	0.00	0.00	0.00	-0.02
	Comp.Mov.	4.01	0.41	9.23	0.00	0.00	0.00	0.01	0.01	0.02
S2	Prop.Mov.	2.31	0.21	6.43	0.00	0.00	0.00	0.00	0.00	0.00
	Comp.Mov.	3.90	0.34	9.22	0.00	0.00	0.00	0.00	0.00	0.00
S3	Prop.Mov.	3.12	0.33	8.42	-0.01	-0.01	0.01	0.00	0.00	0.00
	Comp.Mov.	3.38	0.24	10.13	0.01	0.00	0.02	0.00	0.00	0.00
S4	Prop.Mov.	2.50	0.27	6.53	0.00	0.00	0.00	0.00	0.00	0.00
	Comp.Mov.	2.95	0.24	8.41	0.00	0.00	0.00	0.00	0.00	0.00
S5	Prop.Mov.	2.19	0.22	5.94	0.00	0.00	0.00	0.00	0.00	0.00
	Comp.Mov.	3.67	0.27	11.37	0.00	0.00	0.00	-0.03	-0.02	0.00
**Average**		**2.98**	**0.27**	**8.15**	**0.00**	**0.00**	**0.00**	**0.00**	**0.00**	**0.00**
		**Average relative change**			**0.0 %**	**-0.4 %**	**0.0 %**	**-0.1 %**	**-0.4 %**	**0.0 %**
